# ChatGPT-5 as a leisure health advisor: multidimensional assessment of reliability, quality, usefulness and readability

**DOI:** 10.3389/fpubh.2026.1889768

**Published:** 2026-07-30

**Authors:** Alican Bayram

**Affiliations:** Faculty of Sports Sciences, Bingol University, Bingol, Türkiye

**Keywords:** health communication, large language models, leisure activities, leisure health, quality of health information

## Abstract

**Background:**

The demonstrated protective effects of leisure activities on physical and mental health underscore the need for accessible guidance. Large Language Models (LLMs) like ChatGPT-5 offer a potential solution, yet their application in non-clinical leisure health advice requires rigorous evaluation. This study aims to conduct a multidimensional assessment of ChatGPT-5’s performance in this context.

**Method:**

We generated responses from ChatGPT-5 to 34 common leisure-and-health questions, categorized into six thematic areas (e.g., mental, physical, social health). The responses were assessed using validated evaluation instruments, including the modified DISCERN tool (mDISCERN) to determine reliability, the Global Quality Scale (GQS) to assess overall quality, and a 7-point Likert scale to evaluate perceived usefulness. Readability was assessed using the Flesch Reading Ease (FRE) formula.

**Results:**

ChatGPT-5 demonstrated moderate reliability, good quality, and relatively high usefulness, with mean scores of 3.58/5 for reliability (mDISCERN), 4.11/5 for quality (GQS), and 5.79/7 for usefulness. However, performance varied thematically, with the highest scores in “Leisure and Social Health” and the lowest in personalized contexts like “Age- and Stage-Appropriate Planning.” A critical finding was the low average FRE score of 39, indicating a “difficult” reading level equivalent to U. S. college grades 13–16, which poses a significant accessibility barrier.

**Conclusion:**

While ChatGPT-5 shows promise as a complementary tool for generating leisure health advice, its utility is constrained by suboptimal readability, inconsistent source transparency, and limitations in handling nuanced, personalized scenarios. For safe and effective integration, future developments must prioritize readability optimization, enhanced source citation, and emotional intelligence, all within a framework that emphasizes human oversight.

## Introduction

Engagement in leisure activities has been consistently demonstrated in clinical and epidemiological research to exert protective effects on mental and physical health. Regular participation in such activities not only enhances individual wellbeing but also plays a critical role in the advancement of public health (1). Evidence confirms that regular physical activity undertaken during leisure time reduces the risk of cardiovascular mortality by 19–25% (2) and lowers all-cause mortality (3, 4). Examining 17 different activities, including tennis, running, and gardening, (5) further established the significant contribution of moderate-to-high intensity leisure activities to individual health.

The mental and psychological health dimension of leisure activities constitutes a growing focus of research. These activities provide a robust support mechanism in the contexts of stress management, psychological resilience, meaning-making, and fostering a sense of autonomy (6) Studies have confirmed that nature-based activities (e.g., walking, gardening, mountain sports) are effective in alleviating symptoms of anxiety, depression, and stress, while also supporting mood, cognitive functions, and overall psychological wellbeing (7–9). Although digital transformation has popularized concepts such as “metaleisure” and “e-leisure,” emphasizing virtual and technology-mediated leisure experiences (10), the protective potential of these platforms for physical and mental health warrants careful scrutiny. As digital leisure increasingly shapes how individuals access recreation, health information, and lifestyle guidance, evaluating AI-generated recommendations within these environments has become increasingly important. Recent experimental evidence comparing metaleisure and retro leisure activities in children demonstrated that digitally mediated leisure environments can influence health-related outcomes differently from traditional leisure forms, highlighting the growing need for evidence-based guidance regarding the selection and use of leisure activities in health promotion contexts (11). This rationale informed the inclusion of the “Digital Leisure and Technology Balance” category in the present study. This scrutiny is particularly relevant given that early theoretical work on digital leisure, such as the conceptualization of “electronic idleness” (e-Idle), highlighted its potential links to non-productive consumption and leisure behaviors focused on display rather than wellbeing (12).

The widespread adoption of Large Language Models (LLMs) in the health domain necessitates a systematic evaluation of their accuracy and reliability. Academic perspectives on the role of these models in leisure and health applications are divergent. Sarraju et al. reported that 16% of ChatGPT’s health recommendations were inappropriate and carried potential risks of harm (13), while Shan et al. highlighted that the diagnostic accuracy of LLMs is low compared to medical experts (14). Limitations in clinical use have been associated with low-performing models exhibiting high confidence and minimal differences in confidence levels between correct and incorrect responses (15). Rahsepar Meadi et al. evaluated conversational LLMs as promising tools, albeit with an associated risk of dependency (16), whereas Brown and Tavory pointed to their inadequacies in emotional manipulation and crisis management (17). Zhao and Yuan, while emphasizing risks related to data privacy and algorithmic bias, also noted their potential to enhance global access when used responsibly (18).

Although research on LLMs in the context of leisure remains limited, Barakazi demonstrated their capacity to generate innovative ideas for recreational diversity (e.g., virtual museums, e-sports, AR/VR experiences) (19). However, significant constraints persist, including the provision of outdated information, a lack of emotional understanding, and the risk of hallucination. Bogers et al. drew attention to the multi-query capabilities of LLMs for casual leisure information searches, despite risks of low accuracy and fabrication (20). Wei et al. emphasized the potential for personalized guidance in the tourism sector but underscored the imperative for data verification (21). Evidence from leisure and health contexts indicates that the reliability of LLMs is context-dependent: while expert supervision is essential in clinical applications, their potential as complementary tools exists for non-clinical advice, such as planning leisure activities.

This study is the first to systematically evaluate the performance of ChatGPT-5 in the context of leisure health guidance using four primary metrics: (a) Reliability: verification of information sources using the modified DISCERN instrument; (b) Quality: assessment of content integrity and flow via the Global Quality Scale (GQS); (c) Usefulness: evaluation of practical applicability with an individual-focused 7-point Likert scale; and (d) Readability: clarity analysis employing the Flesch Reading Ease (FRE) formula. This multidimensional assessment addresses a methodological gap in literature, providing an evidence-based framework for understanding the capacity of LLMs in non-clinical domains. Our findings aim to supply critical data for digital health literacy strategies—including verification protocols and readability optimization—thereby guiding the ethical and safe integration of AI-assisted leisure guidance.

## Method

As this study did not include human participants and solely involved the comparison of data generated by computational models with information provided by experts, formal ethics approval was not required.

### Question design

The conceptual design of the study and the development of the 34 leisure-and-health questions were initiated in June 2025. After the study design had been finalized, the finalized question set was entered into the latest version of ChatGPT-5 (OpenAI, San Francisco, CA, USA), and all AI-generated responses were collected on 15 November 2025. Access to ChatGPT-5 was provided through a paid ChatGPT Plus subscription, which was active during the data collection period. To ensure the independence of each response and eliminate potential bias from prior interactions, a new AI session was initiated before addressing each individual question. The prompt dataset consisted of 34 questions that were systematically classified into six predefined thematic categories according to their primary leisure-health objective. The conceptual framework of the prompt dataset is presented in Table 1. The 34 questions were developed by the author of this study, who has doctoral-level expertise in leisure studies and sport sciences. Rather than being directly adopted from a single questionnaire or dataset, the questions were generated by synthesizing the existing literature on leisure and health together with the author’s academic expertise and professional experience in leisure-health planning. The aim was to construct a set of representative, real-world leisure-health questions reflecting the types of information that members of the general public are likely to seek from an AI-based health advisor. Accordingly, the evaluation focused on the usefulness of ChatGPT-5 for members of the general public rather than for clinical patients.

**Table 1:** Thematic framework of the prompt dataset.

Category	Theme	Primary objective	Number of questions	Example prompt
C1	Leisure and Mental Health	Mental relaxation, stress reduction, burnout recovery, attention improvement	6	I get mentally exhausted during the day. Can you create a leisure plan to relax my mind?
C2	Leisure and Physical Health	Physical activity promotion, sedentary behavior reduction, sustainable exercise habits	6	I want a more active lifestyle. What leisure activities could fit my daily routine?
C3	Leisure and Spiritual Health	Inner peace, emotional balance, self-awareness, meaning-making	5	Can you create a leisure plan with activities for my spiritual growth?
C4	Leisure and Social Health	Social participation, relationship building, loneliness reduction	6	I want to strengthen my social relationships. What leisure activities should I join?
C5	Digital Leisure and Technology Balance	Screen time reduction, digital detox, healthy technology use	6	Can you prepare a one-week digital detox leisure plan?
C6	Age- and Stage-Appropriate Healthy Leisure Planning	Personalized leisure planning according to developmental stage and life circumstances	5	Can you suggest fun and developmental leisure activities for my 10-year-old child?

The classification framework was developed to ensure comprehensive coverage of the major dimensions of leisure health while allowing standardized comparisons across thematic domains. The inclusion of Category 5 was informed by emerging concepts such as e-leisure and metaleisure, which describe technology-mediated forms of leisure participation. Accordingly, this category was developed to examine ChatGPT-5’s responses regarding digital recreation, e-leisure participation, and the balance between online and offline leisure activities.

All evaluations, including the application of predefined assessment scales and readability analysis, were conducted by a reviewer (AB) with doctoral-level expertise in leisure studies and sport sciences, including research on health promotion and leisure-health relationships. The reviewer had prior experience using validated health information assessment tools such as mDISCERN and GQS. However, he was not formally trained in health communication or health literacy. The complete set of questions and corresponding ChatGPT-5 responses is provided in Supplementary material 1.

### Reliability evaluation

Information reliability was measured using a modified version of the DISCERN (mDISCERN). Originally developed to evaluate the reliability of written health materials, mDISCERN was adapted to assess the trustworthiness of content generated by artificial intelligence (Table 2). For analysis, responses were coded as 1 for ‘yes’ and 0 for ‘no’ (22, 23).

**Table 2:** Scales used for evaluation of reliability, quality, usefulness, and readability of ChatGPT-5.

Scale, items, and scoring criteria
Modified DISCERN tool
*1 point is given for every yes, with a maximum number of 5 points achievable 1. Are the aims clear and achieved? 2. Are reliable sources of information used? (i.e., publication cited, the responses are from valid studies/sources) 3. Is the information presented by the AI softwares balanced and unbiased? 4. Are additional sources of information listed for patient reference? 5. Does the responses by the AI software address areas of uncertainty?
Global Quality Scale
*scored based on the following characteristics 1. Poor quality, poor flow of the site, most information missing, not at all useful for patients 2. Generally poor quality and poor flow, some information listed but many important topics missing, of very limited use to patients 3. Moderate quality, suboptimal flow, some important information is adequately discussed but others poorly discussed, somewhat useful for patients 4. Good quality and generally good flow. Most of the relevant information is listed, but some topics not covered, useful for patients 5. Excellent quality and flow, very useful for patients
Usefulness Score
*scored based on the following characteristics 1. Not useful at all: Unintelligible language, contradictory information and missing important information. Not useful for patients. 2. Very little useful: Partly clear language is used. Some important information is missing or incorrect. For patients limited use possible. 3. Relatively useful: Clear language is used. Most important information is mentioned, but some important information incomplete or incorrect. 4. Partly useful: Clear language is used. Some important information is missing or incorrect, but most important information is addressed. 5. Moderately useful: Clear language is used and most important information is covered, but some important information is still incomplete or incorrect. 6. Very useful: Clear language is used. All important information is mentioned, but some unimportant information or details are also mentioned. 7. Extremely useful: Clear language is used and all important information is mentioned. Extremely useful to patients, additional information and resources are also provided.
Flesch Reading Ease Reading Level (Estimated reading grade level)
Very easy (US Grade 5 or 11-year-old; 91–100 scores) Easy (US Grade 6; 81–90 scores) Fairly easy (US Grade 7; 71–80 scores) Standard (US Grade 8–9 or 13–15 years; 61–70 scores) Fairly difficult (US Grade 10–12; 51–60 scores) Difficult (US Grade 13–16; 31–50 scores) Very difficult (College graduate; 0–30 scores)

### Quality evaluation

The quality of each response was evaluated using the Global Quality Scale (GQS), a validated tool for evaluating health information (Table 2). Each response was rated on a 5-point scale, where scores of 1–2 reflected low quality, 3 indicated moderate quality, and 4–5 represented high quality (24, 25).

### Usefulness evaluation

Each response was assessed for its usefulness to potential users seeking leisure-health guidance using a ChatGPT-specific usefulness score. This 7-point Likert-type scale measured both clarity and relevance of the content (Table 2). Higher scores represent greater usefulness (26).

### Readability evaluation

Readability was assessed using the Flesch Reading Ease (FRE) formula, a validated and widely adopted tool for evaluating written materials. Developed by Rudolph Flesch in 1948, the FRE score is based on two linguistic components: average sentence length (in words) and average word length (in syllables). Scores range from 0 (very difficult to read) to 100 (very easy to read), with higher values reflecting greater textual simplicity (27).

### Statistical analysis

Statistical analyses were performed using IBM SPSS Statistics version 27 (IBM Corp., Armonk, NY, USA). Each evaluation measure was summarized using descriptive statistics, including indicators of central tendency and distribution, such as the mean, range, minimum, maximum, and mode. Mean scores were also computed for each thematic category: the mDISCERN, GQS, usefulness, and FRE measures.

## Results

**Figure 1:**
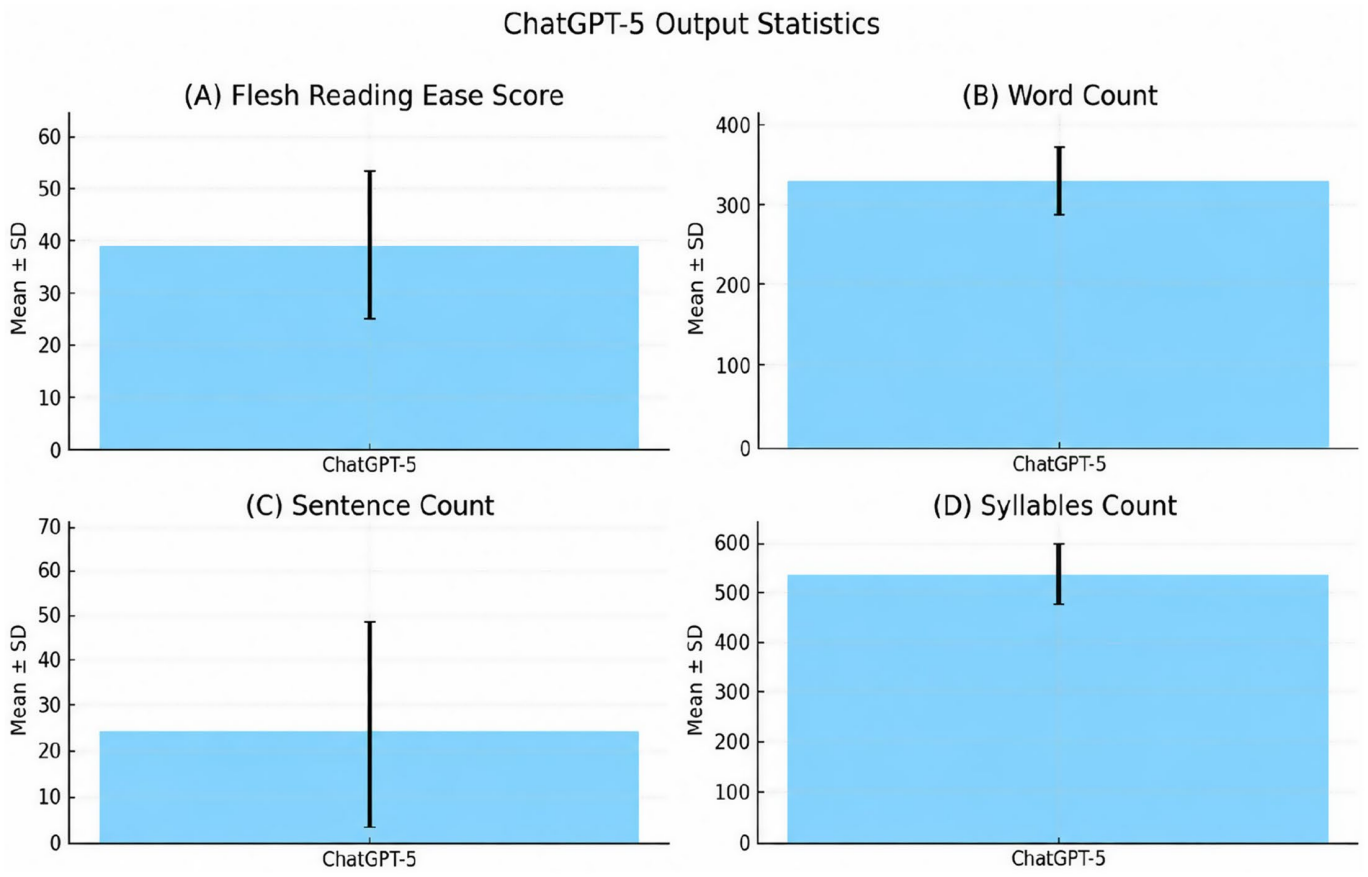
Bar charts show, **(A)** Flesch Reading Ease Score, **(B)** word count, **(C)** sentence count, and **(D)** syllable count for ChatGPT-5.

Mean mDISCERN scores varied across categories, ranging from 3.2 in “Age- and Stage-Appropriate Healthy Leisure Planning” to 4.0 in “Leisure and Spiritual Health,” with an overall average of 3.58. GQS scores were lowest (4.0) in “Leisure and Mental Health,” “Leisure and Physical Health,” and “Age- and Stage-Appropriate Healthy Leisure Planning,” and highest (4.33) in “Leisure and Social Health,” resulting in a mean score of 4.11. Usefulness scores ranged from 5.4 in “Leisure and Spiritual Health” to 6.0 in “Leisure and Physical Health,” “Leisure and Social Health,” and “Age- and Stage-Appropriate Healthy Leisure Planning,” with an overall average of 5.79. FRE values were observed between 35.12 and 43.63, resulting in an average readability score of 39. The highest scores for both quality and usefulness were recorded in “Leisure and Social Health” (GQS: 4.33; Usefulness: 6). In contrast, the lowest values were observed in “Age- and Stage-Appropriate Healthy Leisure Planning” (mDISCERN: 3.2; GQS: 4; Table 3). The average FRE score for ChatGPT-5 was 39, indicating a ‘difficult’ reading level comparable to that of U.S. college students (Grades 13–16). Figure 1 details the word, sentence, and syllable count statistics.

**Table 3:** Reliability, quality, usefulness, and readability level of ChatGPT-5.

Evaluation measure	Descriptive statistic	C1 (*n* = 6)	C2 (*n* = 6)	C3 (*n* = 5)	C4 (*n* = 6)	C5 (*n* = 6)	C6 (*n* = 5)	Total (*n* = 34)
mDISCERN	Mean	3.5	3.5	4	3.83	3.5	3.2	3.58
	Min–Max	3–4	3–4	4–4	3–4	3–4	3–4	3–4
	Range	1	1	0	1	1	1	1
	Mode(s)	3–4	3–4	4	4	3–4	3	4
GQS	Mean	4	4	4.2	4.33	4.16	4	4.11
	Min–Max	3–5	3–5	4–5	4–5	4–5	4–4	3–5
	Range	2	2	1	1	1	0	2
	Mode(s)	4	4	4	4	4	4	4
Usefulness	Mean	5.66	6	5.4	6	5.66	6	5.79
	Min–Max	5–6	5–7	5–6	5–7	5–7	5–7	5–7
	Range	1	2	1	2	2	2	2
	Mode(s)	6	6	5	6	5	6	6
FRE scale	Mean	35.12	36.74	43.09	39.61	43.63	36	39
	Min–Max	12.66–62.44	17.14–47.47	24.11–51.48	31.21–50	15.7–57.9	7.42–69.95	7.42–69.95
	Range	49.78	30.33	27.37	18.79	42.2	62.53	62.53
	Mode(s)	NA	NA	NA	NA	NA	NA	NA

mDISCERN: modified DISCERN, GQS: Global Quality Scale, FRE: Flesch reading ease, C: category; Min: minimum; Max: maximum; C1: leisure and mental health; C2: leisure and physical health; C3: leisure and spiritual health; C4: leisure and social Health; C5: digital leisure – technology balancing; C6: age and stage-appropriate healthy leisure planning.

## Discussion

This study represents the first systematic evaluation of ChatGPT-5’s performance in providing healthy leisure recommendations, assessing it across four key dimensions: reliability, quality, usefulness, and readability. Our principal finding is that while ChatGPT-5 demonstrates significant potential for non-clinical, leisure-related health guidance, its performance is nuanced and varies across thematic categories. The model demonstrated moderate reliability together with good quality and high usefulness, although performance varied across thematic categories. However, the average readability score indicates a “difficult” reading level, comparable to that of U.S. college students. The highest performance was observed in the “Leisure and Social Health” category, while the lowest scores were associated with “Age- and Stage-Appropriate Healthy Leisure Planning.”

The identified readability challenge is a pervasive and critical limitation, consistent with existing literature on LLM-generated health content. For instance, studies evaluating ChatGPT’s responses on topics ranging from hypothyroidism in pregnancy to physical activity guidance for children with cystic fibrosis have consistently reported similarly low readability scores, classifying the text as “difficult” or at a college level (24, 28). Although these previous studies examined readability in specific clinical populations rather than the general public, they consistently demonstrate that LLM-generated health information tends to be written at a reading level that exceeds recommended standards. Unlike those studies, the present study evaluated leisure health advice intended for the general population, whose health literacy levels may be considerably more heterogeneous. Therefore, readability assumes even greater importance in this context, as leisure health recommendations should be readily understandable by individuals with diverse educational backgrounds and varying levels of health literacy. Extending this line of evidence, a recent evaluation of ChatGPT-4’s physical activity counseling for patients with hyperlipidemia reported a mean Flesch Reading Ease score of 40.05, confirming a ‘difficult’ readability level (U.S. college grades 13–16), and further noted that moderate reliability scores were primarily due to the absence of source citations and insufficient guidance for consulting healthcare professionals (29). This issue is further highlighted in evaluations of rheumatological advice and other medical specialties, underscoring that suboptimal readability transcends specific health contexts and populations, including pediatric and adolescent care (24, 30). Low readability poses a significant public health barrier, as complex texts can lead to misunderstanding and misuse, particularly among individuals with lower health literacy. This underscores a key area for improvement, indicating that current LLMs prioritize comprehensiveness and technical accuracy over accessibility. For such tools to be effectively utilized by the public in domains like leisure guidance, future developments must deliberately incorporate readability optimization.

Regarding reliability and quality, our findings align with broader research showing that AI-generated health content often achieves moderate to high scores, yet faces consistent challenges. Previous studies across various medical domains, including patient education materials for chronic diseases and surgical guidance, suggest that while ChatGPT can provide reliable and high-quality information, concerns remain regarding variability, incomplete coverage, and a lack of source citation or transparency (31–34). The lower reliability scores observed in categories requiring personalized, context-specific guidance resonate with findings that ChatGPT may have limitations in providing practical, application-oriented advice compared to more factual, clinical information (28). This indicates that while AI is effective at delivering standard information, its performance may diminish in scenarios requiring nuanced, individualized judgment. Consequently, ChatGPT-5’s capacity to produce structurally sound content for non-clinical leisure guidance is promising, but the absence of transparent source citation and acknowledgment of uncertainties remains a fundamental limitation. This echo concerns raised about health information on other digital platforms, such as YouTube, where similar methodological tools have revealed partially reliable information, necessitating professional verification (25).

The model’s overall usefulness score also varied across categories. High scores in structured, well-defined domains like physical and social health demonstrate its potential to generate practical, actionable content. This aligns with research indicating that perceived usefulness is a key driver of technology adoption (35) and that ChatGPT can excel in providing general recommendations and supportive communication (28). Conversely, the lower score in the more abstract “Leisure and Spiritual Health” category is a critical indicator of AI’s context-dependent utility, reflecting its inherent lack of emotional intelligence and deep contextual understanding, as noted in evaluations across other fields (36, 37). For instance, in response to the question ‘How can leisure activities contribute to meaning in life?’, ChatGPT-5 provided a comprehensive list of potential benefits (e.g., self-reflection, social connection, a sense of purpose) but did not demonstrate an empathic engagement with the deeply personal and subjective nature of meaning-making. The response remained generic and informational, lacking the nuanced, individualized guidance that would characterize a human advisor. This example concretely illustrates the model’s limitations in emotionally and existentially sensitive domains, reinforcing its role as a complementary tool rather than a substitute for human expertise.

Recent findings from a randomized controlled trial indicated that different forms of leisure participation may produce substantially different health outcomes, with traditional leisure activities yielding broader physical fitness benefits than VR-based metaleisure activities among children (11). These findings further underscore the importance of evaluating the quality and reliability of digital leisure guidance systems such as ChatGPT-5. The well-established protective role of leisure for health, increasingly viewed from a critical public and population health perspective as a key domain where individuals can exert control over their lifestyles to manage pervasive time pressure and stress, creates a meaningful application area for LLMs (38) (Mannell, 2007). This is supported by evidence that leisure serves as a direct and indirect resource for psychological, physical, and spiritual wellbeing, with its capacity to act as an effective buffer against stress being particularly pronounced among diverse populations, including those facing socio-economic challenges (39). ChatGPT-5’s demonstrated capacity for generating innovative recreational ideas and structured guidance is consistent with its potential in this domain (19, 21). However, given its limitations in emotionally nuanced areas, variability in responses, and ethical risks such as bias and data privacy (16, 18), its role should be strictly that of a complementary tool. The optimal application likely involves a hybrid model where AI-generated content is blended with human expertise, empathy, and professional oversight to fully leverage the health benefits of leisure while ensuring safety, accuracy, and appropriateness for the individual user.

## Limitations

This study has several limitations that should be considered when interpreting its findings. Firstly, the evaluation was conducted on a single large language model (ChatGPT-5), which restricts the generalizability of the results to other AI models, such as Gemini, Claude, or various local models. A comparative analysis across different LLMs would provide a more comprehensive understanding of the landscape of AI-generated leisure health advice. Secondly, the methodological design relied on assessment by a single reviewer with expertise in the field. The absence of a second independent rater precludes the calculation of inter-rater reliability and introduces the possibility of scoring bias, which is a notable constraint. In addition, although the reviewer had expertise in leisure studies, sport sciences, and health promotion, he was not a specialist in health communication, health literacy, or clinical medicine. Therefore, some evaluations may reflect disciplinary perspectives specific to leisure and health promotion. Future studies should include multidisciplinary reviewers from health communication, public health, and clinical fields. Thirdly, while the study employed validated instruments to assess the content objectively, it did not evaluate how the generated information is perceived, understood, or applied by end-users. The lack of a user experience (UX) focused evaluation means the practical applicability and perceived utility from the perspective of individuals with varying health literacy levels remain unmeasured. Furthermore, the content analysis revealed a critical accessibility barrier: the average Flesch Reading Ease score of 39 indicates a “difficult” reading level, comparable to U.S. college-grade material. This poses a significant challenge for public comprehension, particularly for individuals with lower health literacy, potentially limiting the tool’s real-world effectiveness. Finally, the model’s diminished performance in personalized and nuanced contexts, such as “Age- and Stage-Appropriate Healthy Leisure Planning,” underscores a fundamental limitation in current AI capabilities regarding contextual understanding and emotional intelligence, highlighting areas where human oversight remains indispensable. Additionally, this study did not systematically assess the risk of ‘hallucination’ specifically, the potential for ChatGPT-5 to fabricate sources, generate plausible but incorrect information, or produce references that do not exist. Given that the reliability evaluation (mDISCERN) partially depended on the presence of cited sources, the absence of independent verification of these sources constitutes a significant methodological limitation. Future research should incorporate systematic hallucination detection protocols to validate the factual accuracy of AI-generated leisure health recommendations.

## Conclusion

This study is one of the first comprehensive investigations to systematically evaluate the performance of ChatGPT-5 in the context of leisure health guidance using four primary metrics. The findings reveal that ChatGPT-5 demonstrated moderate reliability, good quality, and relatively high usefulness for non-clinical leisure health advice, with mean scores of 3.58/5 for mDISCERN, 4.11/5 for GQS, and 5.79/7 for usefulness. However, performance differed descriptively across thematic categories. While the highest performance was recorded in structured topics such as “Leisure and Social Health,” the relatively lower reliability and quality scores in areas requiring personalized guidance, like “Age- and Stage-Appropriate Healthy Leisure Planning,” indicate the model’s current limitations in context-specific and nuanced scenarios. One of the most critical findings is that the content generated by the model had an average Flesch Reading Ease score of 39, indicating a “difficult” readability level, which poses a significant barrier to accessibility and comprehensibility, especially for individuals with low health literacy. In conclusion, while ChatGPT-5 shows promising potential as a complementary tool in leisure health consultation when supported by human supervision and expert guidance, it requires improvements in readability, source transparency, and personalization.

### Strengths and weaknesses of the research

The principal strength of this research lies in its pioneering role as the first comprehensive, multidimensional evaluation of ChatGPT-5’s performance specifically within the domain of non-clinical leisure health guidance. By systematically applying four key metrics reliability, quality, usefulness, and readability the study provides a novel, evidence-based framework for assessing LLMs in this context. The methodological rigor is enhanced through the use of validated assessment instruments, including the modified DISCERN tool for reliability, the Global Quality Scale for overall content quality, and a purpose-specific Likert scale for perceived usefulness. Consequently, this work addresses a significant gap in the literature and offers critical foundational data that can inform future digital health literacy strategies, including the development of content verification protocols and readability optimization standards for AI-generated health advice.

However, the research is not without its weaknesses. As noted in the limitations, the scope of this study was restricted to a single AI model, which may limit the generalizability of the findings to other large language models. In addition, all evaluations were conducted by a single reviewer. Although this approach ensured consistency throughout the assessment process, it may have introduced scoring bias and limited inter-rater reliability. Previous studies evaluating AI-generated health information have commonly employed two or more independent reviewers to strengthen methodological rigor and reduce subjective bias. Therefore, the findings of the present study should be interpreted with this consideration in mind. A more robust design would include multiple independent raters and a comparative analysis of various LLMs. Furthermore, the study’s design is inherently analytical rather than experiential; it assesses the intrinsic qualities of the AI output but does not capture the user’s perspective. The absence of a user-centered evaluation means we lack insight into how understandable, trustworthy, or practically helpful the advice is for real people seeking leisure health guidance. This represents a crucial dimension for future investigation to fully appraise the tool’s integration into public health practice.

### Recommendations

Based on the findings of this study, several recommendations are proposed to guide the safe, ethical, and effective development of AI-assisted leisure health guidance. To overcome the identified accessibility barrier, future LLMs should integrate algorithms for automatic readability optimization, aiming to adjust linguistic complexity and simplify jargon to achieve a Flesch Reading Ease score of at least 60 (“Standard” level), thereby making information comprehensible to a broader public. Enhanced source transparency is also vital; models should be designed to provide citations or references for key information and explicitly communicate the limitations or uncertainties inherent in their responses, which would directly address weaknesses in reliability assessment. To improve utility in nuanced scenarios, developmental priorities should include advancing the models’ contextual and emotional intelligence through tailored training data and architectural improvements, enabling more empathetic and personalized guidance, particularly in abstract areas like spiritual health. For the research community, there is a clear need for comparative studies evaluating different LLMs in the leisure guidance context to establish performance benchmarks. Finally, complementing analytical assessments with user-centered research is essential. Future studies should employ UX-focused methodologies to measure real-world users’ perceptions of the reliability, comprehensibility, and practical applicability of AI-generated health advice, ensuring that technological development aligns with public need and understanding.

## Data Availability

The original contributions presented in the study are included in the article/Supplementary material, further inquiries can be directed to the corresponding author.
