# Supplementary material for: ChatGPT-5 as a leisure health advisor: multidimensional assessment of reliability, quality, usefulness and readability
Source: Front Public Health. 2026 Jul 30;14:1889768. doi: 10.3389/fpubh.2026.1889768 (PMC13469005; doi:10.3389/fpubh.2026.1889768)
Supplement: Supplementary file 1 [file Data_Sheet_1.PDF]

No	Q	ChatGPT-5 A
	**Leisure Time and Mental Health**<br><br>In this theme, individuals seek assistance from ChatGPT-5 to plan leisure activities for mental relaxation, stress management, attention improvement, and mental balance.	
1	I get mentally exhausted during the day. Can you create a leisure plan to relax my mind?	**? Weekly Leisure Plan for Mental Relaxation**<br><br>**Monday**<br><br>**Activity:** Guided Meditation (10–15 min)<br>**When:** Evening, after work<br>**Why:** Helps transition your brain from task mode to calm mode. Use free apps like Insight Timer or Calm.<br><br>**Tuesday**<br><br>**Activity:** Nature Walk or Green Space Visit (30 min)<br>**When:** Late afternoon or early evening<br>**Why:** Natural environments reduce cortisol and mental fatigue. Try walking without listening to anything—just observe.<br><br>**Wednesday**<br><br>**Activity:** Light Creative Hobby (Drawing, Journaling, or Music – 30 min)<br>**When:** After dinner<br>**Why:** Engaging in non-demanding creative expression helps process emotions and lowers stress.<br><br>**Thursday**<br><br>**Activity:** Digital Detox Hour + Aromatherapy Bath<br>**When:** One hour before bed<br>**Why:** Reducing screen exposure and stimulating calming senses promotes quality rest and reduces overstimulation.<br><br>**Friday**<br><br>**Activity:** Watch a Comedy or Uplifting Film<br>**When:** Night

		**Why:** Laughter and positive emotions help clear mental tension and restore energy.<br>---<br>**Saturday**<br>**Activity:** Slow Morning with Coffee/Tea + Reading (Fiction or Inspirational – 45 min)<br>**When:** Morning<br>**Why:** A slow start allows the mind to wander and reset without urgency.<br>---<br>**Sunday**<br>**Activity:** Yoga or Stretching + Breathing Exercises (20–30 min)<br>**When:** Early evening<br>**Why:** Helps close the week with mental grounding and body-mind integration.<br>---<br>**Tips:**<br>• Keep your phone away during these activities (unless it's for guided audio).<br>• Don't aim for "productivity"—focus on **presence and restoration**.<br>• Adjust times to fit your real schedule—**consistency beats duration**.
2	I have a stressful life. What leisure activities should I do to feel calmer?	**1. Mind-Body Activities (Calm the Nervous System)**<br>• **Yoga or gentle stretching:** Slows breathing, reduces muscle tension, improves emotional regulation.<br>• **Tai Chi or Qi Gong:** Great for releasing built-up stress in the body while cultivating mental focus.<br>• **Breathwork sessions (10–15 minutes):** Try box breathing or 4-7-8 breathing techniques.<br>---<br>**2. Nature-Based Leisure (Mental Reset & Mood Regulation)**<br>• **Walking in a park, forest, or near water (30–60 mins):** Nature exposure is proven to lower cortisol.<br>• **Gardening or plant care:** Combines light physical activity with soothing focus.

- **Grounding exercises (barefoot walking on grass or sand):** Helps reconnect physically and mentally.

🎨 3. Creative & Sensory Activities (Emotional Expression & Distraction)

- **Adult coloring books or watercolor painting:** Requires focus but not pressure—reduces rumination.
- **Playing a musical instrument or singing softly:** Emotional release and rhythm regulate nervous system.
- **Journaling (especially gratitude or "brain dump" style):** Clears mental clutter and organizes thoughts.

📚 4. Passive Leisure for Emotional Soothing

- **Listening to ambient music, nature sounds, or binaural beats.**
- **Reading fiction, poetry, or calming non-fiction:** Escapism with emotional safety.
- **Watching slow-paced, feel-good shows or documentaries (nature, travel, etc.):** Reduces overstimulation.

---

5. Gentle Social Activities (Support without Pressure)

- **Casual meetups with close friends:** Prefer small, low-energy environments like a café or walk.
- **Volunteering in low-demand roles:** Shifts focus from internal stress to meaningful action.
- **Board games or simple shared hobbies with family or friends:** Connection with light engagement.

Bonus Tip: Micro-Leisure Moments

Even **5–10 minutes** can make a difference. Try:

- Sitting by a window with a warm drink.
- Massaging your hands or neck.
- Aromatherapy (lavender, bergamot, or chamomile oils).

3	I struggle with focus. Can you recommend leisure activities to improve this?	**? 1. Attention Training Activities (Build Concentration Gradually)**<br><br>• **Puzzle-solving (Sudoku, crossword, logic puzzles):** Strengthens sustained attention and problem-solving.<br>• **Board games that require strategy (e.g., chess, Codenames):** Enhances planning, patience, and focus.<br>• **Video games with cognitive elements (e.g., Portal, Monument Valley, Lumosity):** When limited in duration, these can improve visual-spatial attention and reaction time.<br><hr>**🖋 2. Mindful Creative Tasks (Enhance Flow & Presence)**<br><br>• **Drawing or sketching simple objects:** Trains your visual focus and hand-eye coordination.<br>• **Origami or Lego building:** Requires detailed attention and step-by-step sequencing.<br>• **Adult coloring books (especially mandalas):** Stimulates flow state while relaxing your mind.<br><hr>**🌿 3. Nature-Focused Activities (Reset Attention Fatigue)**<br><br>• **Birdwatching or cloud-watching:** Trains sustained and gentle observation.<br>• **Gardening or bonsai care:** Encourages slow focus, tactile interaction, and routine.<br>• **Walking meditation (focusing on breath or footstep rhythm):** Combines mindfulness and movement.<br><hr>**📚 4. Reading for Cognitive Engagement (Strengthen Mental Endurance)**<br><br>• **Start with short stories or essays:** Build up tolerance without overwhelm.<br>• **Practice focused reading (e.g., 20-minute timer, no distractions):** Gradually lengthen your sessions.<br>• **Audiobooks + note-taking:** Combines auditory attention with retention.<br><hr>

<table>
<tr>
<td></td>
<td></td>
<td>

<h3>🎯 5. Attention-Restoring Micro-Habits (Complementary to Leisure)</h3>

Pomodoro walks (5-minute walk after 25 minutes of focus): Clears mental clutter between tasks.
Digital minimalism breaks: Replace screen time with analog hobbies.
Daily “mind warm-up” routines: Brief mindfulness or breathing before your leisure focus task.

<hr>

<h3>Example: Focus-Building Leisure Routine (Compact Daily Format)</h3>

<table>
<tr><th>Day</th><th>Activity</th><th>Duration</th></tr>
<tr><td>Monday</td><td>Coloring mandala + 5-min meditation</td><td>30 min</td></tr>
<tr><td>Tuesday</td><td>Read one short story + summarize</td><td>25 min</td></tr>
<tr><td>Wednesday</td><td>Lego or puzzle building</td><td>40 min</td></tr>
<tr><td>Thursday</td><td>Chess (vs app or friend)</td><td>30 min</td></tr>
<tr><td>Friday</td><td>Garden or plant care + journaling</td><td>30 min</td></tr>
<tr><td>Saturday</td><td>Walking meditation + deep breathing</td><td>45 min</td></tr>
<tr><td>Sunday</td><td>Audio essay + quiet reflection</td><td>30 min</td></tr>
</table>

</td>
</tr>
<tr>
<td>4</td>
<td>I feel burned out. I need leisure suggestions to support my psychological recovery.</td>
<td>

<h3>🌿 1. Sensory Grounding Activities (To Soothe and Regulate)</h3>

These help calm the nervous system and bring you back into your body.

Aromatherapy baths (lavender, eucalyptus, or chamomile oils)
Spending time in nature (sunlight, fresh air, or simply sitting under a tree)
Slow walks without headphones – notice colors, smells, and textures
Listening to ambient or instrumental music with eyes closed

<hr>

</td>
</tr>
</table>

✍ 2. Emotional Offloading & Gentle Expression (To Process Feelings)

Letting thoughts and emotions out in a low-stakes way helps reduce inner tension.

- **Journaling** with prompts like “What do I need today?” or “What’s draining me?”
- **Free-writing sessions** – 10 minutes of uncensored writing
- **Collaging or mood-boarding** your feelings with images or colors
- **Playing an instrument, humming, or singing softly**

---

3. Mind-Body Practices (To Rebuild Inner Calm & Awareness)

These restore internal balance and reduce anxiety associated with burnout.

- **Yoga Nidra** (guided yogic sleep – great for deep mental rest)
- **Gentle yin yoga or restorative yoga** with bolsters and blankets
- **Breathing exercises** (like 4-7-8 or box breathing)
- **Mindfulness meditation** – start with just 5 minutes

🎨 4. Low-Demand Creativity (To Reignite Joy Without Pressure)

These are enjoyable without being mentally taxing or requiring performance.

- **Adult coloring books**
- **Clay modeling or tactile crafts**
- **Embroidery, knitting, or simple DIY crafts**
- **Revisiting childhood hobbies without judgment**

📚 5. Passive Recharge Activities (To Let the Mind Float Freely)

You don't need to "achieve" anything—just be.

- **Reading fiction, poetry, or graphic novels**
- **Watching calm, slow-paced nature documentaries**
- **Listening to comforting podcasts or audiobooks while lying down**
- **Drinking tea in silence while staring out the window**

🗓 Burnout Recovery: Gentle Weekly Plan (Sample)

Day	Activity	Duration
Monday	Journaling + candlelit bath	30–40 min
Tuesday	Outdoor walk + aromatherapy	45 min
Wednesday	Coloring + calm playlist	30 min
Thursday	Guided meditation + tea break	25 min
Friday	Fiction reading + gentle music	40 min
Saturday	Restorative yoga + 10-minute breathwork	45 min
Sunday	Mindful journaling + nature video	30–60 min

✿ A Few Recovery Reminders:

- **You don't need to be productive to be valuable.**
- **Start small**—one activity per day is enough.
- **Permission to rest** is part of healing.

5

Can you create a leisure routine for me to mentally rest and renew myself?

7-Day Leisure Routine for Mental Rest & Renewal

Day 1 – Sunday: Stillness & Silence

- **Activity:** Morning tea/coffee in silence by a window (15–20 min)
- **Evening:** Guided meditation (e.g., "body scan" or "letting go")
- **Goal:** Begin the week by doing less, not more. Let your mind slow down.

Day 2 – Monday: Creative Lightness

- **Activity:** Choose one: adult coloring, sketching, clay work, or journaling (30 min)
- **Bonus:** Play soft instrumental music while creating
- **Goal:** Shift from mental overthinking to hands-on calm expression.

Day 3 – Tuesday: Move with Intention

- **Activity:** Nature walk or slow-paced neighborhood stroll (30–45 min)
- **Evening option:** Light stretching with calm breathing (10 min)
- **Goal:** Reconnect with your body gently and let movement untangle thoughts.

Day 4 – Wednesday: Emotional Unload

- **Activity:** "Brain dump" journaling session (write everything in your head, no filter – 15 min)
- **Followed by:** Deep breathing (e.g., 4-7-8 or box breathing – 5 min)
- **Goal:** Make space in your mind by clearing out mental clutter.

Day 5 – Thursday: Comfort & Nostalgia

- **Activity:** Rewatch a favorite film or show that feels emotionally safe and cozy
- **Pair with:** Your favorite snack or blanket
- **Goal:** Let your brain rest in familiarity—no new input, no effort.

Day 6 – Friday: Presence Over Productivity

- **Activity:** Choose one: mindful cooking, organizing a drawer, or watering plants—do it **slowly**
- **Evening:** 5-min meditation or just lying down with eyes closed and music

<table>
<tr><td></td><td></td><td>
Goal: Enjoy a task without rushing. Train your mind to exist in the moment.
<hr>
<h3>Day 7 – Saturday: Gentle Joy</h3>

Morning: Listen to uplifting or calming music while doing nothing else
Afternoon option: Visit a bookshop, museum, or open space (alone or with a calm companion)
Goal: Renew your sense of curiosity without any pressure to perform.

<hr>
<h3>✿ Optional Daily Anchor (5–10 min):</h3>
Try this each day before bed:

Sit or lie down.
Inhale slowly through the nose (4 sec), hold (4 sec), exhale through the mouth (6 sec).
Reflect on this: “What helped me rest today?”

</td></tr>
<tr><td>6</td><td>I constantly feel tense. Can you suggest relaxing activities I can integrate into my daily life?</td><td>
<h3>☽ Daily Relaxation Activities for Tension Relief</h3>
<h3>□ 1. Morning Grounding Ritual (5–10 min)</h3>

What: Sit quietly with a warm drink and take 10 deep, slow breaths.
Why: Starting the day with calm shifts your body into a relaxed state.
Optional: Place your bare feet on the floor or ground to center yourself.

<hr>
<h3>☘ 2. Nature Micro-Moments (5–20 min)</h3>

What: Step outside, sit by a tree, or look at the sky—even briefly.
Why: Nature exposure lowers cortisol and heart rate—even through a window.

</td></tr>
</table>

- **How to integrate:** Take calls while walking outside, eat near sunlight.

🎧 3. Soothing Sound Breaks (3–15 min)

- **What:** Listen to ambient music, ocean waves, rain sounds, or binaural beats.
- **Why:** Sound vibrations help downshift an overstimulated nervous system.
- **Tip:** Use these during transitions—after work, before bed, or mid-day.

✍ 4. Mental Unloading with Journaling (10 min)

- **What:** Write down what's stressing you—or use prompts like "What do I need right now?"
- **Why:** Naming tension reduces its grip. Writing helps process and release it.
- **When:** Before bed or after a stressful task.

🛁 5. Evening Wind-Down Ritual (15–30 min)

- **Options:**
  - Aromatherapy bath or warm shower with slow breathing
  - Candlelit room + herbal tea + soft instrumental music
  - Reading fiction (not news or work-related content)
- **Why:** Signal to your body that it's time to shift into calm and rest.

💆 6. Body Tension Reset (2–5 min anytime)

- **Try:** Progressive Muscle Relaxation (tighten and release one body part at a time)
- **Or:** 10 shoulder rolls + jaw stretch + deep exhale
- **Why:** Releases stored tension from your body instantly.

📵 7. Digital Detox Moments (10–30 min/day)

- **What:** Put your phone away—no notifications—for even 10 minutes.
- **Pair with:** Doing nothing, or any of the above activities.
- **Why:** Tension often builds from overstimulation and alerts. Your brain needs stillness.

Sample Daily Relaxation Flow

Time	Activity	Duration
Morning	Deep breathing + warm drink	10 min
Midday	Nature walk or calming music break	15 min
Afternoon	Tension release stretch or journaling	10 min
Evening	Bath + book or candlelight + soft music	30 min
Anytime	5 deep breaths with hand on chest	1 min

Leisure Time and Physical Health

In this theme, individuals seek assistance from ChatGPT-5 to plan leisure activities for maintaining physical health, developing an active lifestyle, and combating sedentary behavior.

7

I want a more active lifestyle. What leisure activities could fit my daily routine?

🏃‍♀️ Daily-Friendly Active Leisure Activities

1. Walking with Intention

- **How:** Morning, lunch, or evening walks (15–30 min)
- **Why:** Easy to integrate, clears your mind, boosts cardiovascular health.
- **Try:** Walk-and-talk calls, podcasts, or "audiobook walks."

---

2. Dance Sessions (Solo or Group)

- **How:** 10–20 minutes of dancing to music you love
- **Why:** Boosts mood, burns calories, and feels more like fun than fitness.
- **Try:** YouTube dance workouts or just freestyle in your living room.

---

3. Bike Rides (Leisure or Commute)

- **How:** Ride around your neighborhood, to run errands, or explore new areas
- **Why:** Improves stamina and offers a sense of adventure.
- **Bonus:** Low-impact on joints compared to running.

---

4. Active Video Games or VR

- **How:** Use platforms like Ring Fit Adventure, Just Dance, or VR boxing
- **Why:** Blends movement with entertainment—great for staying consistent.

---

5. Recreational Sports or Classes

- **Options:**
  - Pick-up basketball, tennis, or table tennis
  - Martial arts, climbing, or group dance/yoga classes
  - Swimming (great for full-body low-impact activity)
- **Why:** Social, motivating, and often more enjoyable than solo exercise.

---

6. Stretch-and-Move Breaks During the Day

- **How:** Set a timer to stretch, do squats, or walk every 60–90 minutes
- **Why:** Prevents stiffness and improves circulation during sedentary days.
- **Tip:** Combine with music to make it feel like a mini ritual.

---

7. Outdoor Exploration Activities

- **Ideas:**
  - Hiking trails or nature reserves on weekends
  - Urban exploring on foot or by bike
  - Geocaching or nature photography walks
- **Why:** Keeps you active while feeding curiosity and play.

		🗓 Sample Active Leisure Routine (Flexible)<br><br>**Time** / **Activity** / **Duration**<br>Morning / 10-min stretch or walk / 10 min<br>Midday / Dance to 2 songs or walk break / 10–15 min<br>Evening / Bike ride or active video game / 20–30 min<br>Weekend / Recreational sport or hike / 60+ min<br><br>---<br><br>*🔄 Tips to Keep It Sustainable:*<br><br>• **Start small and celebrate consistency**, not intensity.<br>• **Mix structured and playful movement** to avoid burnout.<br>• **Use music, apps, or accountability partners** to boost motivation.
8	I'm too sedentary, and it bothers me. What leisure suggestions do you have to maintain my physical health?	*Active Leisure Suggestions for Sedentary Lifestyle Recovery*<br><br>**🚶‍♂️ 1. Intentional Daily Walking**<br><br>• **What:** 2–3 short walks per day (10–15 min each)<br>• **Why:** Walking boosts circulation, burns calories, supports joint health, and reduces the risks of inactivity.<br>• **How to start:** Take phone calls while walking, walk after meals, or park farther away.<br><br>---<br><br>**2. Beginner-Friendly Stretching or Mobility Routines**<br><br>• **What:** Morning or evening stretch (5–10 min)<br>• **Why:** Loosens stiff muscles, improves posture, and activates the body gently.<br>• **Resources:** Search YouTube for "5-minute mobility" or "desk stretch routine"<br><br>---<br><br>**💃 3. Dancing as Movement Play**<br><br>• **What:** Put on a playlist and move freely (10–20 min)

- **Why:** Elevates heart rate, boosts mood, and doesn't feel like a chore.
- **Tip:** Do it while cleaning, cooking, or on breaks from work.

🎮 4. Active Gaming or VR Activities

- **What:** Try motion-based games (e.g., Ring Fit Adventure, Just Dance, Beat Saber)
- **Why:** Fun way to sweat without needing equipment or leaving home.

---

5. Physically Engaging Hobbies

- **Options:**
  - Gardening
  - DIY projects or rearranging home spaces
  - Playing catch or frisbee
  - Light hiking or birdwatching in local parks
- **Why:** These keep you active while doing something meaningful or creative.

🏓 6. Low-Pressure Recreational Sports

- **Options:** Table tennis, pickleball, badminton, swimming, bowling
- **Why:** Gentle on the body but improves agility, coordination, and heart health.

---

7. "Functional Activity" Integration

- **Idea:** Treat everyday tasks as mini-movement sessions
  - Do a few squats while brushing teeth
  - Walk while listening to podcasts
  - Clean with music and dance moves
- **Why:** These add up without taking extra time.

<table>
<tr>
<td></td>
<td></td>
<td>

🗓️ *Sample Daily Movement Integration Plan*

Time	Activity	Duration
Morning	Light stretching or 5-min yoga	5–10 min
Midday	Short walk after lunch	10–15 min
Evening	Dance break, walk, or active hobby	20–30 min
Total Daily	Light, enjoyable movement	30–45 min

🔄 Make It Stick:

- **Attach movement to routines you already do** (after meals, during calls)
- **Track your daily movement** for motivation—not perfection
- **Choose activities you enjoy**, not ones that feel like punishment

</td>
</tr>
<tr>
<td>9</td>
<td>Can you plan fun leisure activities that incorporate exercise?</td>
<td>

🎉 *7-Day Fun & Active Leisure Plan*

Monday – Dance Party Solo

- **Activity:** Put on your favorite upbeat playlist and dance like nobody's watching
- **Duration:** 20–30 minutes
- **Benefits:** Cardio, coordination, endorphins
- **Fun Tip:** Use disco lights or mirror effects for extra energy!

Tuesday – Outdoor Photo Walk

- **Activity:** Go for a 30-minute walk and take photos of interesting things (architecture, plants, shadows)
- **Benefits:** Low-impact cardio, mindfulness, creative stimulation
- **Fun Tip:** Try a "color hunt" – find and photograph 5 things of one color.

Wednesday – Mini Games Night (Active Edition)

</td>
</tr>
</table>

- **Activity:** Play interactive games like:
    - Charades
    - Twister
    - Dance-based video games (Just Dance, Beat Saber)
- **Duration:** 30–45 minutes
- **Benefits:** Movement + laughter = stress relief + physical activity

---

Thursday – Nature Adventure

- **Activity:** Go to a nearby park, beach, or forest trail for walking or light hiking
- **Duration:** 45–60 minutes
- **Benefits:** Endurance, mental clarity, vitamin D
- **Fun Tip:** Bring a friend, picnic, or podcast to enjoy along the way.

Friday – Sporty Social Night

- **Activity:** Invite friends or family for:
    - Pickleball
    - Mini-golf
    - Bowling
    - Badminton
- **Duration:** 1–2 hours
- **Benefits:** Light competition, movement, connection
- **Fun Tip:** Keep it playful—no score stress!

Saturday – “Try Something New” Day

- **Activity:** Try a new physical hobby:
    - Rock climbing
    - Zumba class
    - Roller skating
    - Aqua fitness
- **Duration:** Varies (usually 45–60 min)
- **Benefits:** Novelty boosts dopamine and keeps fitness fresh

---

Sunday – Active Recovery & Play

- **Activity:** Choose something gentle but engaging:

<table>
<tr><td></td><td></td><td>

Yoga in the park
Frisbee with friends
Stretching while watching a movie

Duration: 20–40 minutes
Benefits: Relaxation + circulation without strain

<hr>
<h3>🗓️ Customize It Further:</h3>
Would you prefer:

Indoor-only options?
Solo vs social activities?
10–15 minute versions for busy days?

</td></tr>
<tr><td>10</td><td>What leisure activities should I do regularly to stay healthy?</td><td>
<h3>🧘‍♀️ 1. Mind-Body Activities (for Stress Relief & Mental Clarity)</h3>

Yoga or stretching (2–3x/week): Increases flexibility, reduces stress hormones, and supports emotional regulation.
Meditation or breathwork (daily, even 5–10 min): Sharpens focus and improves resilience to stress.
Tai Chi or Qigong (1–2x/week): Low-impact movement that strengthens balance, focus, and calm.

<hr>
<h3>🏃‍♂️ 2. Light-to-Moderate Physical Activities (for Heart & Body Health)</h3>

Brisk walking, cycling, or swimming (3–5x/week): Improves cardiovascular health, mood, and metabolism.
Dancing, hiking, or active sports (1–2x/week): Fun ways to keep the body moving and burn calories.
Active chores or gardening (most days): Everyday tasks that count toward physical activity.

<hr>
<h3>🎨 3. Creative Activities (for Emotional Wellness & Brain Health)</h3>

Art, music, or craftwork (1–2x/week): Stimulates creativity and reduces anxiety.

</td></tr>
</table>

- **Writing or journaling (2–3x/week):** Enhances emotional clarity and reflection.
- **Playing a musical instrument or singing:** Supports brain health and self-expression.

---

4. Nature-Based Leisure (for Immune & Mental Boosts)

- **Spending time in green spaces (2–3x/week):** Lowers blood pressure, reduces inflammation, and enhances mood.
- **Forest walks or gardening:** Combines physical and emotional benefits.

---

5. Social Leisure (for Longevity & Happiness)

- **Quality time with friends or loved ones (weekly):** Reduces risk of depression and strengthens immune response.
- **Group hobbies or clubs:** Offers belonging, accountability, and purpose.
- **Volunteering (even monthly):** Improves self-esteem and life satisfaction.

---

6. Mental Stimulation (for Cognitive Health)

- **Reading, puzzles, or games (2–4x/week):** Supports memory, focus, and neuroplasticity.
- **Learning a new skill or hobby:** Keeps the brain adaptable and curious.

Weekly Healthy Leisure Blueprint Example

Day	Activity	Category
Monday	30-min walk + evening journaling	Physical + Creative
Tuesday	Stretching + short social phone call	Mind-Body + Social
Wednesday	Gardening or housework + reading	Physical + Mental

		Thursday	Yoga class or dancing at home	Mind-Body + Physical
		Friday	Art or music + nature walk	Creative + Nature
		Saturday	Hike or sport + social hangout	Physical + Social
		Sunday	Meditation + puzzle game + family time	Mind-Body + Cognitive + Social

---

✅ Key to Making It Stick:

- Choose what **feels enjoyable**, not forced.
- Start with **small durations** and build consistency.
- Mix up solo and social, indoor and outdoor options.

11	I struggle with exercise. What alternative leisure activities can I try?	*Alternative Leisure Activities to Traditional Exercise*

🌿 Alternative Leisure Activities to Traditional Exercise

💃 1. Dancing (Even Alone at Home)

- **Why it works:** It's fun, expressive, and great for cardiovascular health.
- **How to start:** Put on music you love and move however you want.
- **Bonus:** Dance for just 10–15 minutes a day—it adds up.

🚶 2. Purposeful Walking

- **Ideas:**
  - Walk to run errands
  - Take short strolls while listening to music or a podcast
  - Explore new parks or neighborhoods
- **Why it works:** It lowers stress, burns calories, and improves circulation—without feeling like a "workout."

🎮 3. Active Video Games or VR

- **Options:** Just Dance, Ring Fit Adventure, Beat Saber, or Wii Sports

- **Why it works:** Blends play with movement. It feels like a game, not exercise.

---

4. Gentle Movement Practices

- **Try:**
  - Yoga
  - Tai Chi
  - Stretching with calming music
- **Why it works:** Enhances flexibility, body awareness, and relaxation.

---

5. Active Home Tasks

- **Ideas:**
  - Cleaning or reorganizing while listening to music
  - Gardening or planting herbs
  - Rearranging furniture or doing DIY projects
- **Why it works:** You're moving without realizing you're “exercising.”

🏓 6. Casual Recreational Activities

- **Fun & friendly options:**
  - Table tennis
  - Mini golf
  - Bowling
  - Light badminton
- **Why it works:** Involves movement, but feels like social play.

🐕 7. Animal-Assisted Activity

- **Ideas:**
  - Walk or play with a dog
  - Visit a pet café or animal shelter
- **Why it works:** Adds joy and movement together, especially if you're more emotionally than physically motivated.

<table>
<tr>
<td></td>
<td></td>
<td>

🌼 *Mental Health + Movement Bonus:*

- **Art walks** (visit galleries or murals)
- **Photography walks** (capture textures, colors, or themes)
- **Slow, mindful walking** in nature (no phone, just presence)

🗓️ Gentle Weekly Example Plan

Day	Activity	Duration
Monday	Dance to 3 songs	10–15 min
Tuesday	Organize your space + stretching	20 min
Wednesday	Walk to café with a book	30 min
Thursday	Yoga or gentle flow video	20 min
Friday	Active video game	30 min
Saturday	Visit park or market on foot	45 min
Sunday	Pet playtime or creative hobby	30 min

</td>
</tr>
<tr>
<td>12</td>
<td>I want to improve my stamina. Can you prepare a low-intensity but sustainable leisure plan?</td>
<td>

💪 *7-Day Low-Intensity Stamina-Building Leisure Plan*

🗓️ Day 1 – Slow & Steady Walk

- **Activity:** 20–30 minute brisk walk (outdoors or on a treadmill)
- **Bonus:** Listen to a podcast or audiobook while walking.
- **Goal:** Begin activating your cardiovascular system with low impact.

🗓️ Day 2 – Gentle Yoga or Stretch & Flow

- **Activity:** Follow a beginner yoga video or do a self-paced full-body stretch (20–25 min)
- **Goal:** Improve circulation, joint mobility, and breathing control—key for stamina.

</td>
</tr>
</table>

📅 Day 3 – Active Chores + Light Dance

- **Activity:** Do 20–30 minutes of active home tasks (e.g., vacuuming, organizing, mopping)
- **Add:** 10 minutes of light dancing to your favorite upbeat music
- **Goal:** Keep your body in motion without formal exercise.

📅 Day 4 – Nature Walk or Park Exploration

- **Activity:** Visit a local park, garden, or trail for a 30–45 minute relaxed walk
- **Tip:** Include mild elevation or stairs if possible
- **Goal:** Add variety and mild endurance challenge through terrain.

📅 Day 5 – Active Hobby or Creative Movement

- **Activity:** Choose one:
  - Gardening
  - Playing an instrument while standing
  - Doing art/crafts while walking around or standing
- **Duration:** 30–45 minutes
- **Goal:** Light, sustained movement + creative engagement = mental + physical endurance.

📅 Day 6 – Low-Impact Cardio Option

- **Activity Options:**
  - Swimming or aqua walking
  - Cycling on low resistance
  - Active video games like Ring Fit Adventure or Wii Sports
- **Duration:** 25–35 minutes
- **Goal:** Gradual heart rate elevation while preserving joint comfort.

📅 Day 7 – Mindful Movement + Breathwork

<table>
<tr>
<td></td>
<td></td>
<td>

- **Activity:** 15-minute walk or stretch + 10 minutes of breathing (e.g., box breathing or 4-7-8 technique)
- **Goal:** End the week with integration—calm focus and controlled stamina building.

📌 *Weekly Summary Overview*

<table>
<tr><th>Day</th><th>Focus</th><th>Duration</th></tr>
<tr><td>Monday</td><td>Walking + audio content</td><td>30 min</td></tr>
<tr><td>Tuesday</td><td>Yoga/stretch</td><td>25 min</td></tr>
<tr><td>Wednesday</td><td>Chores + light dancing</td><td>30–40 min</td></tr>
<tr><td>Thursday</td><td>Outdoor relaxed walk</td><td>40 min</td></tr>
<tr><td>Friday</td><td>Hobby with light movement</td><td>30–45 min</td></tr>
<tr><td>Saturday</td><td>Light cardio play</td><td>25–35 min</td></tr>
<tr><td>Sunday</td><td>Breath + stretch</td><td>25 min</td></tr>
</table>

✅ Tips for Building Stamina Gently:

- **Pace yourself**—aim to feel energized *after*, not exhausted.
- **Focus on duration, not intensity** at first.
- **Hydrate and rest** well—recovery helps build stamina too.
- Track your progress (time, steps, or how you *feel*) once a week to stay motivated.

</td>
</tr>
<tr>
<td></td>
<td colspan="2">

Leisure Time and Spiritual Health

*In this theme, individuals seek assistance from ChatGPT-5 to plan leisure activities for gaining inner peace, emotional balance, meaning-seeking, and spiritual wholeness.

</td>
</tr>
<tr>
<td>13</td>
<td>I feel emotionally drained. What leisure activities can balance my mood?</td>
<td>

🌼 *Leisure Activities to Balance Your Mood*

□♀ 1. Restorative Mind-Body Practices (Regulate the Nervous System)

- **Gentle yoga or stretching with music** (10–30 min)
- **Breathing exercises** (4-7-8, box breathing, or simply slow exhales)
- **Guided meditations focused on emotional grounding**

</td>
</tr>
</table>

- **Why:** These reduce cortisol, improve emotional regulation, and slow racing thoughts.

🎨 2. Creative Expression (Process Feelings Without Words)

- **Painting, coloring, or doodling freely** – no skill needed
- **Playing soft music, singing, or improvising sounds**
- **Journaling prompts like:** “What do I need right now?” or “Today I feel…”
- **Why:** Creativity allows emotional release without explanation.

🌿 3. Nature-Based Reconnection (Soothe and Reset)

- **Sit in a green space, even briefly**
- **Walk barefoot on grass or sand (grounding)**
- **Tend to plants or create a mini garden**
- **Why:** Natural environments lower blood pressure and stabilize mood.

🖥 4. Comfort & Nostalgia-Based Activities (Emotional Safety)

- **Rewatch a feel-good movie or series from your past**
- **Flip through old photos or listen to childhood music**
- **Do a hobby you loved as a kid (e.g., puzzles, Legos, stickers)**
- **Why:** Familiarity creates emotional safety and calms the brain.

□ 5. Gentle Social Connection (Emotional Containment)

- **Talk to someone who listens without fixing**
- **Low-energy social activities:** tea with a friend, silent companionship, or walking together
- **Online support groups or journaling communities**
- **Why:** Connection—without pressure—helps regulate emotion.

☕ 6. Mini Self-Soothing Rituals (Daily Mood Maintenance)

- **Tea or cocoa break with no phone**
- **Lighting a candle and reading a few pages of fiction**
- **Aromatherapy (lavender, orange, or rose)**
- **Hot bath with calming music**
- **Why:** Short moments of peace throughout the day build emotional resilience.

🗓️ *Sample 3-Day Mood-Balancing Routine*

Day	Morning	Afternoon	Evening
Day 1	5-min breathwork	Nature walk (20 min)	Journal + candlelight bath
Day 2	Comfort music + tea	Creative activity (coloring)	Watch nostalgic film
Day 3	Gentle yoga (10 min)	Gratitude journaling (5 min)	Chat or walk with a trusted friend

---

💡 Final Tips:

- You don't need to "fix" everything—just start with *one* small soothing activity.
- **Mood shifts happen slowly**, so be patient and consistent.
- **Avoid stimulation overload**—choose soft, slow, sensory-rich experiences.

14 | I want to feel more at peace and explore myself. How should I plan my leisure time for this purpose?

🌿 *Guiding Principles for Peaceful, Reflective Leisure*

1. **Slow down** – peace needs space and time. Choose unhurried, quiet moments.
2. **Be intentional** – make time to tune into yourself, not just pass time.
3. **Balance solitude and softness** – allow yourself to feel, reflect, and simply *be*.
4. **Prioritize depth over entertainment** – choose meaningful over mindless.

🧘‍♀️Weekly Leisure Plan for Inner Peace & Self-Discovery

📅 Day 1 – Stillness & Silence

- **Morning:** Sit quietly with tea or coffee (no phone) – 10 min
- **Evening:** Guided meditation (body scan or "being with yourself") – 15 min
- **Purpose:** Create space for your inner voice to emerge

📅 Day 2 – Reflective Journaling

- **Activity:** Journal with prompts like:
    - "What does peace mean to me?"
    - "When do I feel most like myself?"
- **Bonus:** Add soft instrumental music
- **Purpose:** Process thoughts and discover patterns

📅 Day 3 – Mindful Movement

- **Activity:** Gentle yoga, stretching, or tai chi – 20–30 min
- **Option:** Practice barefoot in nature or a quiet room
- **Purpose:** Align body and mind through slow, intentional movement

---

📅 Day 4 – Nature as a Mirror

- **Activity:** Silent walk in nature (park, beach, trail) – 30–60 min
- **Try:** No music. Just observe and breathe.
- **Purpose:** Let nature ground you and reflect your state of mind

---

📅 Day 5 – Creative Self-Expression

- **Activity:** Choose one:
    - Watercolor painting
    - Collaging a "mood board" of your inner world
    - Improvising on a musical instrument

- **Purpose:** Let your subconscious speak through form, color, and sound

---

🗓 Day 6 – Restorative Rituals

- **Activity:** Aromatherapy bath, tea ceremony, or candlelit breathing
- **Add:** Soothing textures, scents, and quiet music
- **Purpose:** Regulate your nervous system and honor your inner calm

---

🗓 Day 7 – Soulful Reading or Listening

- **Activity:** Read or listen to content that inspires deep thought (e.g., Rumi, Eckhart Tolle, Thich Nhat Hanh, poetry)
- **Reflect:** "What part of this resonated with me?"
- **Purpose:** Connect with timeless wisdom and your own inner truths

---

🌙 *Optional Daily Anchor (5–10 min)*

- **Evening check-in:** Ask yourself:
  "What did I notice about myself today?"
  "What do I need to feel more whole tomorrow?"

💡 *Final Tips:*

- Create a **peaceful space** at home—light, scent, sound, and comfort matter.
- **Be gentle with yourself**—inner peace is not a goal but a practice.
- Avoid multitasking—**presence is the path to peace**.

15	I'd like leisure suggestions that increase emotional awareness and nourish me.	*🌿 Leisure Suggestions for Emotional Awareness & Nourishment*<br><br>**✍ 1. Reflective Journaling**<br><br>• **How:** Write freely, or use prompts like:<br>○ "What am I feeling right now?"<br>○ "What is this emotion trying to teach me?"<br>○ "What part of me needs kindness today?"<br>• **Why:** Journaling helps you name emotions, uncover patterns, and access insight.<br><br>---<br><br>**🎨 2. Emotional Art or Visual Journaling**<br><br>• **How:** Express how you feel using colors, symbols, or abstract shapes—no need for skill.<br>• **Try:** Mood mandalas, collage-making, or "draw your day" sketches<br>• **Why:** Non-verbal expression helps you externalize what's hard to articulate.<br><br>---<br><br>**🎧 3. Music That Mirrors or Shifts Mood**<br><br>• **How:** Create playlists:<br>○ One that reflects your current emotion (for release)<br>○ One that lifts or calms you (for shift)<br>• **Why:** Music bypasses logic and connects directly to feeling.<br><br>---<br><br>**□ 4. Somatic Awareness Practices**<br><br>• **Try:**<br>○ Body scans (noticing where tension or emotion sits)<br>○ Gentle yoga focused on hips, chest, and shoulders (where emotions are often held)<br>• **Why:** Emotions often live in the body. Movement can help you feel and release them safely.<br><br>---<br><br>**💬 5. Voice & Expression Practices**

- **How:**
    - Read poetry aloud that resonates with you
    - Speak your emotions in a private voice memo
    - Sing softly—especially emotional or meaningful songs
- **Why:** Speaking or vocalizing gives emotions a path outward and increases self-recognition.

🌳 6. Mindful Walks with Emotional Curiosity

- **How:** Walk slowly and ask:
    - “What am I carrying today?”
    - “What does this landscape reflect in me?”
- **Why:** Nature provides containment, clarity, and metaphor for what we feel.

📚 7. Emotionally Resonant Reading

- **What to read:** Memoirs, reflective essays, gentle psychology books, or poetry (e.g., Rupi Kaur, Mary Oliver)
- **Why:** Connecting with others’ emotional journeys helps normalize and validate your own.

🛁 8. Sensory Self-Compassion Rituals

- **Ideas:**
    - Take a long bath with soothing scents
    - Apply lotion slowly and lovingly to your skin
    - Prepare a warm, nourishing meal and eat without distractions
- **Why:** Touch, scent, and warmth all send your nervous system messages of safety and worthiness.

🗓️ *Example: 3-Day Emotional Nourishment Flow*

Day	Activity	Time
Day 1	Emotion journaling + soft playlist	Evening
Day 2	Body scan + visual art (no words)	Afternoon

		Day 3 Nature walk + poetry aloud Morning<br><br>---<br><br>💡 *Tips for Emotional Awareness Through Leisure*<br><br>• **Go slow.** Emotions often speak softly at first.<br>• **Let it be messy.** Awareness doesn't require control.<br>• **Follow what *feels* honest.** Your emotional truth is a compass, not a problem.
16	I want to rebuild myself. What supportive leisure activities could help in this process?	🌱 *Supportive Leisure Activities for Rebuilding the Self*<br><br>---<br><br>**✍ 1. Identity Journaling (Who Am I Now?)**<br><br>• **Try prompts like:**<br>○ "What parts of me feel tired? What parts are waking up?"<br>○ "What do I value now that I didn't before?"<br>○ "What does rebuilding look like—for me?"<br>• **Why:** Helps you rediscover your inner landscape and make sense of change.<br><br>---<br><br>**2. Grounding Mind-Body Practices (Build Stability from Within)**<br><br>• **Try:**<br>○ Slow yoga (focus on rooting poses like mountain, tree, or child's pose)<br>○ Breathwork (like box breathing or long exhales)<br>○ Body scanning meditation (bring awareness back to your center)<br>• **Why:** These practices restore inner balance and self-trust, step by step.<br><br>---<br><br>**🎨 3. Creative Play Without Expectation**<br><br>• **Options:**<br>○ Watercolor, collage, clay, or creative journaling<br>○ "Self-portrait" art projects (abstract or symbolic)

- **Why:** Rebuilding happens through re-creation. Art helps you express parts of yourself beyond words.

🌳 4. Nature Immersion for Clarity and Symbolism

- **Activities:**
  - Silent walks in a natural place (observe what draws your attention)
  - Sit under a tree and reflect: "What is growing in me now?"
  - Collect items (stones, leaves) that symbolize release or new beginnings
- **Why:** Nature mirrors life cycles and offers wisdom without judgment.

💬 5. Story & Reflection through Books, Podcasts, or Memoirs

- **Choose content about:**
  - Resilience
  - Transformation
  - Personal healing
- **Why:** Hearing others' journeys helps you feel less alone—and inspires hope.

🛠 6. Personal Rebuilding Projects

- **Examples:**
  - Decluttering or redesigning a space to reflect who you are now
  - Creating a vision board or life map
  - Learning a new skill or hobby (reclaiming curiosity)
- **Why:** These external acts of building echo and support internal reconstruction.

---

7. Gentle Social Rituals

- **Try:**
  - Regular walks with someone who sees you
  - Sharing creative projects with a trusted friend

- Writing letters (to past self, future self, or someone meaningful)

- **Why:** Rebuilding doesn't have to be lonely. Safe connection reinforces your new foundation.

🗓 *Sample 5-Day "Rebuilding You" Leisure Routine*

Day	Focus	Activity
Day 1	Grounding	Gentle yoga + identity journaling (20–30 min)
Day 2	Expression	Visual journaling (colors, symbols, feelings)
Day 3	Clarity	Nature walk + reflection question
Day 4	Visioning	Create a vision board or "new self" list
Day 5	Integration	Self-compassion bath + podcast or book

🌟 *Gentle Reminders as You Rebuild:*

- **You're not starting from scratch—you're starting from experience.**
- **You can grow in soft ways.** Strength doesn't require force.
- **Self-rebuilding is a form of art, not a checklist.**

17 Can you create a leisure plan with activities for my spiritual growth?

🌿 *7-Day Leisure Plan for Spiritual Growth*

🗓 Day 1 – Create Sacred Space

Activity: Set up a simple altar or reflection corner
What to include: A candle, natural object (stone, flower), photo, or meaningful text
Ritual: Sit quietly near it for 10 minutes; breathe and say, "I'm here."
Purpose: Establishes intention, stillness, and a sacred rhythm

---

🗓 Day 2 – Silence & Stillness

Activity: Practice 15 minutes of silent sitting or breath awareness
Optional: Focus on your breath or repeat a grounding word (e.g.,

“peace” or “now”)
Purpose: Cultivates presence and makes space for the subtle voice within

---

🗓 Day 3 – Reflective Journaling

Prompt ideas:

- “When do I feel most alive or connected?”
- “What guides me when I feel lost?”
- “What does spiritual growth mean to me?”
  Purpose: Deepens inner clarity and opens dialogue with your true self

---

🗓 Day 4 – Nature as Teacher

Activity: Take a mindful nature walk (30–60 min)
How: No music, no phone—just presence. Observe, breathe, receive.
Reflect: What qualities of nature do I want to grow in myself (e.g., resilience, flow)?
Purpose: Connects you to something larger and timeless

🗓 Day 5 – Spiritual Reading & Reflection

Activity: Read something soulful or sacred (e.g., Rumi, Kahlil Gibran, Mary Oliver, Tao Te Ching)
How: Read slowly; pause when a phrase moves you.
Write down or speak: What line spoke to me and why?
Purpose: Receives wisdom and strengthens your spiritual vocabulary

---

🗓 Day 6 – Creative Offering

Activity: Create something as a devotional or expressive act
Options:

- Paint a feeling or prayer
- Write a poem or a letter to the universe

		• Build a small nature mandala<br>**Purpose:** Uses leisure as spiritual expression<br><br>---<br><br>**Day 7 – Gratitude & Letting Go Ritual**<br><br>**Activity:** Light a candle and write:<br><br>• 3 things you're grateful for<br>• 1 thing you want to release or surrender<br>**Optional:** Burn the paper safely or bury it<br>**Purpose:** Completes the week with reflection, reverence, and release<br><br>---<br><br>*Daily Anchor (Optional)*<br><br>At any point in your day, pause for 1 minute and silently ask:<br><br>“What is sacred about this moment?”<br><br>---<br><br>*Tips for Sustained Spiritual Leisure*<br><br>• You don’t need to “feel” spiritual—just **show up gently and honestly**<br>• Repeat this 7-day plan or mix & match what resonates<br>• Let quietness and mystery be part of your practice
	**Leisure Time and Social Health**<br><br>In this theme, individuals seek assistance from ChatGPT-5 to plan leisure activities for strengthening social relationships, reducing loneliness, increasing social participation, and enhancing community bonds.	
18	I want to strengthen my social relationships. What types of leisure activities should I join?	*Leisure Activities to Strengthen Social Bonds*<br><br>**1. Collaborative Games & Challenges**<br><br>• **What to try:**<br>○ Board game nights<br>○ Puzzle-solving or escape rooms<br>○ Trivia or game-show-style nights

- **Why:** Friendly competition and teamwork build trust and laughter

---

🏃 2. Group Movement Activities

- **What to join:**
  - Walking clubs or hiking groups
  - Recreational sports (like pickleball, volleyball, or casual soccer)
  - Dance or yoga classes
- **Why:** Moving together builds emotional synchrony and shared rhythm

---

🎨 3. Creative Gatherings

- **What to do:**
  - Attend or host a paint night, poetry reading, or music jam
  - Group journaling, storytelling, or collage workshops
- **Why:** Sharing creativity invites vulnerability and expression

---

☐ 4. Small Social Rituals

- **Ideas:**
  - Weekly coffee or tea meetups
  - "Soup and story" nights (each person brings a story and dish)
  - Evening walks with a friend
- **Why:** Simple rituals become emotional anchors over time

🌿 5. Volunteer Together

- **Where to go:**
  - Community gardens, animal shelters, food banks
  - Organize group donation or care package events
- **Why:** Helping others together creates a sense of shared purpose and bonding

---

🎭 6. Shared Learning Experiences

- **Options:**
  - Language classes
  - Pottery, photography, or cooking workshops
  - Book clubs or film discussion groups
- **Why:** Learning side-by-side creates connection through curiosity and growth

---

📅 7. Seasonal or Cultural Events

- **Examples:**
  - Attend local festivals, markets, or fairs
  - Celebrate cultural holidays with others
  - Host seasonal potlucks or outdoor picnics
- **Why:** Shared celebration fosters unity and joy

📆 Sample Weekly Social Leisure Plan

Day	Activity	Social Benefit
Monday	Group walk with a friend	Relaxed, one-on-one bonding
Wednesday	Cooking night with family or friends	Collaboration & joy
Friday	Attend a community event or game	Group energy & discovery
Sunday	Creative circle (art, stories, tea)	Intimate sharing & support

---

💡 Final Tips:

- **Consistency matters more than intensity**—make small, regular social plans
- Choose people you **feel safe and authentic with**
- Don't wait for invites—**initiate low-pressure hangouts** like "walk & talk" or "coffee & journaling"

19	I want to meet more people. How should I spend my leisure time?	🌍 *Leisure Strategies for Meeting New People*<br>---<br>**🎯 1. Join Interest-Based Groups or Clubs**<br>• **What to try:**<br>○ Book clubs<br>○ Board game cafés<br>○ Walking/hiking groups<br>○ Language exchange meetups<br>• **Why it works:** Shared passions = instant conversation starters<br>---<br>**🎨 2. Attend Creative or Skill-Based Workshops**<br>• **Options:**<br>○ Pottery, painting, cooking, or photography classes<br>○ Dance or theater workshops<br>○ Writing or journaling circles<br>• **Why it works:** Everyone's learning together, so social walls drop<br>---<br>**□♀ 3. Join Mind-Body or Wellness Communities**<br>• **Ideas:**<br>○ Yoga, tai chi, or meditation classes with group reflection<br>○ Mindful walking or nature therapy meetups<br>• **Why it works:** These spaces often attract reflective, open-minded people<br>---<br>**□ 4. Attend Community Events & Food-Based Gatherings**<br>• **Examples:**<br>○ Farmers' markets, open mic nights, food truck festivals<br>○ "Meet & eat" events or cultural dinners<br>• **Why it works:** Food and music naturally spark interaction<br>---

🏓 5. Try Light Team Sports or Movement Clubs

- **Low-pressure sports:**
  - Pickleball
  - Bowling
  - Recreational volleyball or dance socials
- **Why it works:** Movement reduces awkwardness and builds camaraderie

---

🐾 6. Participate in Shared Volunteering

- **Where to go:**
  - Animal shelters
  - Beach or park cleanups
  - Community events or charity runs
- **Why it works:** Helping others = meaningful, natural connection

---

🖥💻 7. Use Leisure-Focused Social Apps (In Real Life)

- **Apps like:**
  - Meetup
  - Bumble BFF
  - Eventbrite (local events)
- **Tip:** Look for group activities, not 1-on-1 pressure

🗓 Sample Weekly Leisure Plan to Meet People

Day	Activity	Setting Type
Monday	Attend a yoga or group fitness class	Wellness + social
Wednesday	Join a book club or art night	Creative + reflective
Friday	Go to a community event or open mic	Casual + cultural
Saturday	Volunteer in a group (e.g., local garden)	Purpose-driven
Sunday	Go to a group hike or coffee meetup	Nature + relaxed

		💬 *Tips for Building Connection While Out*<br><br>• **Make eye contact and smile**—open body language invites connection<br>• Ask open questions like: *“What brought you here?”* or *“Have you done this before?”*<br>• **Follow up** with people you vibe with—invite them to a second shared activity
20	Can you suggest socially-oriented leisure activities to reduce my loneliness?	*Leisure Activities to Reduce Loneliness Through Social Connection*<br><br>☕ **1. Join a Regular Meetup Group**<br><br>• **Types to explore:**<br>○ Book clubs<br>○ Language exchange meetups<br>○ Walking or running groups<br>• **Why it helps:** Consistent groups create familiarity and belonging over time.<br><br>---<br><br>🎨 **2. Attend a Creative Workshop or Hobby Circle**<br><br>• **Options:**<br>○ Pottery, drawing, collage nights<br>○ Music jam sessions or open mic sign-ups<br>• **Why it helps:** Creating side by side invites connection *without pressure to talk constantly*.<br><br>---<br><br>🐾 **3. Volunteer With a Purpose**<br><br>• **Places to help:**<br>○ Animal shelters (walking dogs = instant companionship)<br>○ Food banks<br>○ Local libraries or art centers<br>• **Why it helps:** You’ll bond with others over shared values and feel part of something meaningful.<br><br>---

4. Join Group Wellness Activities

- **Ideas:**
  - Gentle yoga or tai chi classes
  - Meditation groups with post-practice sharing
- **Why it helps:** A calm space to feel connected spiritually and emotionally—perfect for introverts.

🎭 5. Get Involved in Local Theater or Improv

- **Even backstage counts!** You could help with costumes, props, or lighting.
- **Why it helps:** Theater builds fast, tight-knit communities and gives you a role to play—literally and socially.

🍲 6. Try Communal Meals or Potlucks

- **What to try:**
  - Cultural potlucks
  - Community center dinners
  - Cooking classes with shared meals
- **Why it helps:** Sharing food breaks down barriers and invites conversation naturally.

📚 7. Attend Storytelling or Conversation Events

- **Examples:**
  - Story circles
  - TEDx salon events
  - "Human Library" or peer-dialogue sessions
- **Why it helps:** Deep listening and authentic stories reduce emotional isolation.

🗓 Sample 5-Day "Anti-Loneliness" Leisure Plan

Day	Activity	Social Style
Monday	Volunteer at local animal shelter	Purpose + presence
Tuesday	Attend a book discussion group	Casual + engaging

		Thursday Join an art journaling or paint night Creative + quiet<br>Friday Share a meal at a community dinner Nourishing + open<br>Sunday Go to a group meditation + tea reflection Calm + bonding<br>---<br>*Gentle Tips to Help You Feel More Connected:*<br>• **Start small and show up consistently**—even if just once a week<br>• Focus on **shared activity** rather than forced conversation<br>• Don't be afraid to say, *"This is my first time here"*—it invites welcome
21	I want to spend time with family/friends. What activities could we do together?	🏡 *Relaxed & Cozy At-Home Activities*<br>🎲 **1. Game Night**<br>• **Options:** Board games, charades, trivia, or card games<br>• **Why it works:** Encourages laughter, teamwork, and playful competition<br>🎬 **2. Movie or Series Marathon**<br>• **Theme ideas:** Childhood favorites, foreign films, comedy nights<br>• **Add:** Popcorn, discussion afterward<br>• **Why it works:** Shared viewing = emotional bonding<br>🍳 **3. Cook Together & Share a Meal**<br>• **Ideas:** Try a recipe from a different culture, DIY pizza night, or a taco bar<br>• **Why it works:** Food brings people together and turns chores into joy<br>🎨 **4. Creative Craft Session**<br>• **Options:** Paint night, DIY candles, collage, memory scrapbooks<br>• **Why it works:** Side-by-side creativity opens up conversation organically<br>---

		🌳 Outdoor & Active Activities<br><br>**🚶‍♂️ 5. Nature Walks or Picnics**<br>• **Where:** Parks, riversides, botanical gardens<br>• **Add:** Bring a frisbee, cards, or a speaker for music<br>• **Why it works:** Nature + no screens = quality connection<br><br>**🏞 6. Day Trips or Local Adventures**<br>• **Ideas:** Explore nearby towns, hiking trails, farmers' markets, or museums<br>• **Why it works:** Novelty + shared exploration = lasting memories<br><br>**🚲 7. Bike Rides or Group Strolls**<br>• **Add:** Choose scenic or nostalgic routes<br>• **Why it works:** Movement side-by-side encourages natural conversation<br><br>---<br><br>*🕊 Meaningful & Emotional Activities*<br><br>**📷 8. Look Through Old Photos or Home Videos**<br>• **Why it works:** Sparks storytelling, laughter, and intergenerational bonding<br><br>**💬 9. Memory Sharing or Story Circles**<br>• **Prompt ideas:**<br>○ “Tell a story from a trip we took.”<br>○ “What’s a time we all laughed the hardest?”<br>• **Why it works:** Creates intimacy and appreciation<br><br>---<br><br>*🌟 Bonus: Monthly Rituals to Keep the Bond Strong*<br><br>• **Family movie Sundays**<br>• **Friday dinner rotations (everyone picks the menu once a month)**<br>• **Monthly “friendship walks” or coffee catchups**<br>• **Joint hobby project (like gardening or a shared playlist)**

<table>
<tr>
<td></td>
<td></td>
<td>
<hr>
📅Sample Weekly Bonding Plan
<table>
<tr><th>Day</th><th>Activity</th><th>Who with</th></tr>
<tr><td>Monday</td><td>Cook a meal together + eat mindfully</td><td>Family or housemates</td></tr>
<tr><td>Wednesday</td><td>Short walk or call with a close friend</td><td>Local or distant pal</td></tr>
<tr><td>Friday</td><td>Board game or movie night</td><td>Small group</td></tr>
<tr><td>Sunday</td><td>Nature outing or visit a museum</td><td>Family or mixed group</td></tr>
</table>
</td>
</tr>
<tr>
<td>22</td>
<td>Can you plan leisure activities to develop my social skills?</td>
<td>
Leisure Plan to Develop Social Skills
<hr>
🗣 1. Attend Conversation-Based Meetups

Examples:

Language exchange nights
Philosophy cafés or “big questions” meetups
Book clubs with discussion segments

Skill built: Articulating thoughts, active listening, respectful dialogue

<hr>
🎭 2. Try Improv, Acting, or Public Speaking Groups

Join:

Local improv workshops
Toastmasters or public storytelling events
Drama classes (even just for fun)

Skill built: Thinking on your feet, projecting confidence, reading cues

<hr>
3. Join a Collaborative Hobby Group

Ideas:

Community gardening
Group cooking classes
Music jams or collaborative art nights

Skill built: Cooperative communication, casual chatting, initiating conversations

</td>
</tr>
</table>

☕ 4. Practice One-on-One Connection

- **Activity:** Invite someone for coffee, a walk, or co-working in a café
- **Tip:** Prepare 2–3 thoughtful questions (e.g., "What do you love doing outside of work?")
- **Skill built:** Emotional presence, curiosity, conversation flow

---

🎲 5. Join Social Game Nights

- **Games to try:**
  - Codenames, Pictionary, Werewolf, or even trivia nights
- **Skill built:** Reading group dynamics, turn-taking, playful interaction

---

6. Attend Mindfulness-Based Group Activities

- **Try:**
  - Group meditation or yoga with sharing circles
  - Breathwork + reflection sessions
- **Skill built:** Self-awareness, emotional expression, group attunement

---

📅 *Weekly Social Skills Leisure Schedule (Sample)*

Day	Activity	Focused Skill
Monday	Attend a book club or discussion group	Listening + response
Wednesday	Improv or public speaking practice	Spontaneity + confidence
Friday	Join a social game night	Social play + adaptability
Saturday	1-on-1 coffee or hobby with a new acquaintance	Deepening connection
Sunday	Group yoga/meditation + short reflection chat	Presence + emotional attunement

		---<br><br>*🌱 Extra Practice Tips*<br><br>• After each activity, reflect:<br>○ "What went well?"<br>○ "What felt challenging?"<br>○ "What will I try next time?"<br>• Don't aim for perfection—**aim for presence** and growth<br>• Smile, ask open-ended questions, and express genuine interest in others
23	Could you recommend group activities or hobbies that encourage socialization?	*🎨 Creative & Expressive Hobbies (Conversation While Creating)*<br><br>**1. Art Nights or Craft Workshops**<br><br>• **Try:** Painting, pottery, collage, jewelry-making<br>• **Why it works:** Casual, hands-on activity opens room for side-by-side conversation<br><br>**2. Creative Writing or Journaling Groups**<br><br>• **Try:** Writing prompts, zine making, poetry-sharing circles<br>• **Why it works:** Expressive, reflective, and emotionally bonding<br><br>---<br><br>*🧘‍♀️ Wellness & Mind-Body Activities (Shared Calm & Presence)*<br><br>**3. Group Yoga or Meditation Classes**<br><br>• Especially those with a **post-practice tea or sharing circle**<br><br>**4. Walking or Nature Therapy Groups**<br><br>• Mindful hikes, forest bathing, or just community walking clubs<br>• Great for natural conversation without eye contact pressure<br><br>---

□ Games & Strategy (Fun with Friendly Competition)

5. Board Game Cafés or Clubs

- Games like Codenames, Dixit, Catan, or cooperative card games
- Encourages teamwork, laughter, and natural interaction

6. Trivia Nights or Pub Quizzes

- Join with a team or attend solo and team up with strangers
- Easy way to bond over shared knowledge and fun

🏓 Active & Movement-Based Hobbies (Social Through Play)

7. Recreational Sports Leagues

- Try volleyball, pickleball, frisbee, or bowling leagues
- Low-stakes sports build camaraderie through movement

8. Dance Classes or Social Dances

- Salsa, swing, line dancing, or even ecstatic dance
- Encourages fun physical connection + casual conversation

📚 Shared Learning Experiences (Connect Through Curiosity)

9. Language Classes or Language Exchange Groups

- Everyone's learning—great equalizer for beginners

10. Cooking Classes

- Often include group work, tasting, and shared laughter

🌱 Community & Purpose-Driven Activities (Bond Through Meaning)

11. Volunteering Groups

- Community gardens, food banks, animal shelters

		• Connects you with kind people through service<br>**12. Cultural or Spiritual Discussion Circles**<br>• Book-based philosophy nights, spiritual meetups, or cultural exchange events<br>---<br>🎭 *Performance & Storytelling (Express, Listen, Laugh)*<br>**13. Theater Groups or Improv Classes**<br>• Build confidence, quick thinking, and deep bonding<br>**14. Story Circles or Human Library Events**<br>• Spaces to share real-life stories and practice listening deeply
	**Digital Leisure – Technology Balancing**<br>In this theme, individuals seek assistance from ChatGPT-5 to plan leisure activities for reducing screen time, coping with digital fatigue, and maintaining a balanced relationship with technology.	
24	I'm constantly looking at screens. What leisure activities can I do for a more balanced life?	🌿 *Screen-Free Leisure Activities for a More Balanced Life*<br>---<br>✍ **1. Journaling & Reflection**<br>• **How to start:**<br>○ Morning pages (freewriting for 10 minutes)<br>○ Gratitude lists<br>○ Reflective prompts like: "What did I enjoy today that didn't involve a screen?"<br>• **Why it helps:** Brings clarity and slows mental noise<br>---<br>🌳 **2. Nature-Based Activities**<br>• **Try:**<br>○ Forest walks or hikes<br>○ Gardening or caring for houseplants<br>○ Cloud watching or star gazing

- **Why it helps:** Nature restores attention, lowers stress, and reconnects you to your senses

---

🎨 3. Creative Hobbies (No Screens Allowed)

- **Ideas:**
  - Drawing, painting, collage
  - Knitting, embroidery, or woodwork
  - DIY home decor or crafts
- **Why it helps:** Tactile, slow-paced creativity invites flow and calm

---

📚 4. Deep Reading (With Real Books!)

- **Start a ritual:** Read 15–30 minutes each morning or night
- **Bonus:** Join an in-person book club for connection
- **Why it helps:** Rebuilds focus and imagination away from digital distractions

---

5. Mind-Body Practices

- **Screen-free options:**
  - Yoga or stretching with no music
  - Breathwork or candlelight meditation
  - Qi Gong or mindful walking
- **Why it helps:** Regulates nervous system and restores present-moment awareness

---

🎲 6. Analog Play & Games

- **What to play:**
  - Card games, board games, jigsaw puzzles
  - Paper-based brain games (crosswords, sudoku)
- **Try:** Weekly game night with friends or family
- **Why it helps:** Brings joy, mental challenge, and social bonding

---

✉ 7. Letter Writing or Scrapbooking

- **Write to:** Friends, future self, or loved ones
- **Scrapbook ideas:** A "screen-free joy" journal or memory album
- **Why it helps:** Fosters connection and analog nostalgia

🍵 8. Slow Living Rituals

- **Ideas:**
  - Tea-making as a mindful moment
  - Slow cooking (bread baking, soups, stews)
  - Setting a cozy mood with candles and silence
- **Why it helps:** Shifts your body and mind into a restful rhythm

---

🗓 *Sample Screen-Free Daily Rhythm*

Time of Day	Screen-Free Activity	Duration
Morning	Journal + tea near a window	15 min
Afternoon	Nature walk or hands-on hobby	30–60 min
Evening	Read a physical book or write a letter	30 min
Before Bed	Gentle stretching + quiet breath practice	10–15 min

---

✨ *Final Tips:*

- Create a **tech-free zone or hour** daily
- Use **physical clocks, timers, or journals** to avoid phone use
- **Start with one screen-free block per day** and build from there

25 I want quality time beyond digital games. What alternative leisure activities do you suggest?

🎲 *Fun & Interactive Group Activities (Social + Playful)*

1. Board Game Nights (Analog Style)

- **Try:** Codenames, Dixit, Wingspan, Azul, or cooperative games like Pandemic

- **Why:** Brings out fun, strategy, and face-to-face interaction

2. Puzzle Sessions

- Jigsaw puzzles or logic puzzles with music and snacks
- Great for relaxing together while staying mentally engaged

3. Murder Mystery or Role-Play Nights

- Use printable kits or make your own
- Encourages creativity, collaboration, and laughter

🎨 *Creative Hands-On Hobbies (Self-Expression + Flow)*

4. Painting, Drawing, or Collage Making

- Try themed sessions like “paint your mood” or “paint with a twist”

5. Craft Nights or DIY Projects

- Candle-making, origami, scrapbooking, or upcycling clothes/furniture
- Good for solo or group creativity

🌿 *Outdoor Leisure (Movement + Fresh Air)*

6. Nature Walks, Hikes, or Urban Explorations

- Discover local trails, nature reserves, or architectural walks

7. Group Bike Rides or Picnics

- Bring games (frisbee, cards), food, and leave screens behind

---

🍳 *Food-Based Bonding (Tastes + Togetherness)*

8. Cook or Bake Together

- Try a new recipe or recreate a family favorite

- Option: Have a “cooking challenge” using mystery ingredients

9. Host a Theme Dinner or Cultural Food Night

- Learn and cook dishes from a different country each time

📚 Mindful & Reflective Options (Depth + Stillness)

10. Book Club or Reading Aloud Sessions

- Take turns reading passages from a novel, poetry, or essays
- Discuss with hot tea or wine for a cozy vibe

11. Journaling or Storytelling Circles

- Use prompts like “a memory I cherish…” or “the best trip I’ve taken”
- Great for sharing and learning more about each other

🧘‍♀️ Restorative Rituals (Peace + Presence)

12. Group Yoga, Stretching, or Meditation

- Even 15–20 minutes before dinner can bring calm and connection

13. Evening Rituals: Candlelight Talks or Gratitude Circles

- Sit together, light a candle, and each person shares one thing they’re grateful for

---

🌟 Bonus: “Digital-Free Day” Challenge

Pick one day per week where you all commit to screen-free leisure:

- Plan meals, walks, games, and creative time
- Reflect at the end: *“What did I enjoy most about today?”*

26	Can you create a leisure plan to balance my technology use?	📵 *7-Day Leisure Plan to Balance Technology Use*<br>---<br>🗓️ **Day 1 – Digital Awareness + Journaling**<br>**Activity:**<br>• Track your screen time (honestly)<br>• Journal: *"When do I use screens the most—and why?"*<br>• Set 1 clear intention (e.g., no phone during meals or before bed)<br>**Why:** Awareness is the first step toward balance.<br>---<br>🗓️ **Day 2 – Move Without Screens**<br>**Activity:**<br>• Go for a walk, hike, or bike ride **without headphones or a phone**<br>• Invite a friend or go solo—just be present<br>**Why:** Movement clears mental fog and reconnects you with your body and surroundings.<br>---<br>🗓️ **Day 3 – Create with Your Hands**<br>**Activity Options:**<br>• Painting, baking, gardening, or journaling<br>• Try a puzzle or DIY craft<br>• Keep your phone in another room<br>**Why:** Hands-on leisure anchors you in the present moment.<br>---<br>🗓️ **Day 4 – Connect in Real Life**<br>**Activity:**<br>• Make plans with someone offline: coffee, cooking, game night, or walk

- If no one's available, write a letter or call someone

Why: True connection often comes from face-to-face or voice-to-voice presence.

🗓 Day 5 – Digital Sunset

Activity:

- Shut down all screens at least 1 hour before bedtime
- Use that time for: reading, gentle stretching, meditation, or reflection

Why: Protects your sleep and resets your overstimulated brain.

🗓 Day 6 – Screen-Free Mini Retreat (Half-Day)

Activity:

- Spend half the day without any screens. Suggestions:
  - Go to the park or library
  - Cook slowly
  - Write, draw, or rest
  - Reflect on how you feel

Why: Time away reboots your nervous system and restores attention span.

🗓 Day 7 – Reset & Recalibrate

Activity:

- Reflect: *What did I miss about screens? What did I love without them?*
- Decide on new habits to keep (e.g., no phone during meals, screen-free mornings)

Why: Integration is key—leisure is about sustainability, not restriction.

		🍁 *Optional Daily Anchors*<br><br>**Time of Day** — **Screen-Free Habit Idea**<br>Morning — Stretch + journal (no phone yet)<br>Midday break — 10-min outdoor walk (no devices)<br>Dinner — Eat tech-free, with music or talk<br>Before bed — Read, meditate, or reflect offline
27	I'm looking for enjoyable activities outside the virtual world. What do you recommend?	🌿 *Enjoyable Activities Outside the Virtual World*<br><br>---<br>🎨 **Creative & Hands-On Joy**<br>• **Painting, collage, or watercolor journaling**<br>• **Pottery or sculpting with clay**<br>• **DIY crafts** (e.g., making candles, beading, upcycling clothes)<br>• **Creative writing** (poems, short stories, gratitude lists)<br><br>**Why:** Making things with your hands engages your senses and slows your mind.<br><br>---<br>🌳 **Nature & Outdoor Recharge**<br>• **Walk or hike in a new park or trail**<br>• **Sit under a tree and read or journal**<br>• **Picnic with a friend or solo**<br>• **Gardening or planting herbs on a balcony**<br>• **Birdwatching or cloud-gazing**<br><br>**Why:** Nature helps reset your brain and uplifts your mood—no Wi-Fi needed.<br><br>---<br>□ **Playful Movement & Fun**<br>• **Dance in your room to music you love**<br>• **Join a group fitness, dance, or yoga class**<br>• **Rollerblading, biking, or jumping rope**<br>• **Revisit childhood games**: hula hoop, hopscotch, hacky sack

Why: Movement releases endorphins and connects you to your body.

🎲 Social & Group Activities

- **Game nights with friends** (board games, cards, trivia)
- **Cooking together**—try a new dish or themed dinner
- **Attend local events** (markets, festivals, craft fairs)
- **Join a club or group** (book club, walking group, art circle)

Why: Real-life laughter and shared moments ease loneliness and recharge connection.

📚 Solo Serenity & Growth

- **Read physical books**—fiction, memoir, or self-reflection
- **Write letters to loved ones or to your future self**
- **Try meditation or breathwork** with no app—just silence
- **Visit museums, libraries, or botanical gardens**

Why: Deep, quiet joy often comes from simply being with yourself.

Adventurous & Unusual Ideas

- **Take a local train to a town you've never explored**
- **Visit a flea market and hunt for treasures**
- **Try something completely new—archery? paddleboarding? improv class?**
- **Volunteer at a shelter, garden, or food pantry**

Why: Novelty activates your curiosity and makes you feel alive.

🗓 *Sample "Offline Joy" Weekend Plan*

Time	Activity	Setting
Saturday AM	Hike or walk in nature	Outdoor
Saturday PM	Art or DIY project	Home or studio
Sunday AM	Farmers' market + cooking lunch	Local

<table>
<tr><td></td><td></td><td>Sunday PM   Read on a blanket in the park   Outdoor</td></tr>
<tr><td>28</td><td>Can you plan physical/mental leisure activities to reduce my digital fatigue?</td><td>

📵 *7-Day Anti-Digital Fatigue Leisure Plan*

📅 Day 1 – Ground & Breathe (Mental Reset)

Activity: 15–20 minutes of mindful breathing + nature time

- Find a quiet spot outdoors (park, backyard, balcony)
- Practice slow breathing: inhale 4 sec, exhale 6 sec
- Focus on sounds, smells, colors around you

✅ *Benefit:* Restores attention, lowers nervous system overload

---

📅 Day 2 – Move with Joy (Physical Reboot)

Activity: Dance, stretch, or yoga flow to music (no screens)

- Let go of rules—just move freely
- Try: 10–30 minutes of gentle or playful movement

✅ *Benefit:* Releases tension stored from screen posture + boosts endorphins

---

📅 Day 3 – Analog Creativity (Mental Flow)

Activity: Hands-on creative hobby (screen-free)

- Try: sketching, watercolors, collage, or journaling
- Bonus: Write 1 page on "How I feel when I unplug"

✅ *Benefit:* Activates deep focus and rewires scattered attention

---

📅 Day 4 – Connection Time (Emotional Recharge)

Activity: Meaningful time with a loved one

- Share a meal, walk, or play a board game together
- Phone stays on airplane mode

</td></tr>
</table>

✅ *Benefit:* Replaces digital connection with emotional presence

---

🗓 Day 5 – Silent Stimulation (Mental Rest)

Activity: Solo walk in silence (no podcast, no music)

- Focus on sounds, pace, breath, and your feet on the ground
- 20–45 minutes is ideal

✅ *Benefit:* Gives your mind a rare break from input overload

---

🗓 Day 6 – Physical Play (Body Activation)

Activity: Choose a joyful physical activity:

- Frisbee, nature hike, group sport, biking, even climbing stairs
- Do it outdoors for bonus effects

✅ *Benefit:* Counteracts sedentary screen time + builds body-mind connection

---

🗓 Day 7 – Reflect + Design Your "Tech-Lite" Future

Activity:

- Journal: "What's one habit I want to change?"
- Design a weekly screen-free ritual (e.g., Sunday hike, no-phone dinners)
- Plan 1 recurring analog joy (e.g., book club, game night, yoga group)

✅ *Benefit:* Long-term mindset shift toward healthier tech-life balance

---

? *Optional Daily Anchors (Mental Hygiene Boost)*

Time	Anchor Habit (No Screen Required)
Morning	Stretch + set intention aloud

		Midday 5-minute breathing pause<br><br>Evening Read a physical book or write a reflection<br><br>Bedtime Soft light, silence, or music—no phone
29	I want a digital detox. Can you prepare a one-week plan for me?	📵 *One-Week Digital Detox Plan*<br><br>□ *Goal:* Rest your mind, reset your habits, and reconnect with real life.<br>📅 *Format:* Each day includes a focus theme, offline activities, and a simple intention.<br><br>---<br><br>◆ **Day 1 – Awareness & Intention**<br><br>**Theme:** Observe + Prepare<br>**Digital Rule:** Track your screen time without judgment<br>**Activities:**<br><br>• Journal: "When and why do I use screens the most?"<br>• Create a "digital-free zone" at home (e.g., dining table, bedroom)<br>• Choose 3 favorite offline activities to focus on this week<br><br>✅ *Intention:* "I'm becoming aware of what drains and restores me."<br><br>---<br><br>◆ **Day 2 – Move Your Body, Not Your Feed**<br><br>**Theme:** Physical Activation<br>**Digital Rule:** No screens during physical activity<br>**Activities:**<br><br>• Go for a long walk, hike, or try a dance/yoga session<br>• Do one active chore mindfully (cleaning, gardening, biking)<br>• Stretch or do breathwork before bed<br><br>✅ *Intention:* "My body is alive and deserves my attention."<br><br>---<br><br>◆ **Day 3 – Create, Don't Scroll**

Theme: Hands-On Creativity
Digital Rule: No phone or laptop for 4+ hours during the day
Activities:

- Draw, write, cook, bake, build something, or collage
- Start a journal or scrapbook for this detox journey
- Visit a bookstore, market, or museum—just observe

✅ *Intention:* "I create more than I consume."

◆ Day 4 – Connect Offline

Theme: Real Conversations
Digital Rule: No texting or social media—make 1 real-life connection
Activities:

- Call a friend or have an in-person chat
- Write a letter or postcard to someone
- Share a meal without any screens nearby

✅ *Intention:* "I connect in ways that matter."

◆ Day 5 – Digital-Free Morning

Theme: Mindful Start
Digital Rule: No screens for the first 2 hours after waking
Activities:

- Start your day with tea/coffee and a book or notebook
- Take a tech-free walk or do light exercise
- Reflect: "How did this morning feel different?"

✅ *Intention:* "I deserve a calm beginning to each day."

◆ Day 6 – The Silent Half-Day

Theme: Mental Rest
Digital Rule: No screens for a full half-day (4–6 hours)
Activities:

- Be still: try meditation, nature time, or napping

		• Avoid music, TV, podcasts—just silence or natural sound<br>• Do nothing—and observe how that feels<br><br>✅ *Intention:* "Silence nourishes me more than noise."<br><br>---<br><br>**◆ Day 7 – Rebuild Intentionally**<br><br>**Theme:** Reflection & Integration<br>**Digital Rule:** Use screens only for essentials<br>**Activities:**<br><br>• Journal: "What did I learn? What do I want to keep?"<br>• Design 2–3 weekly habits: e.g., screen-free Sundays, phone-free dinners<br>• Celebrate with a screen-free meal, walk, or bath<br><br>✅ *Intention:* "I can choose how I use technology."<br><br>---<br><br>*Optional Add-Ons:*<br><br>• 📘 Read a physical book this week<br>• 🎲 Host a board game or card night<br>• 🌳 Spend 30+ minutes in nature each day<br>• ✍ Use a notebook instead of a note-taking app
	**Age and Life Stage-Appropriate Healthy Leisure Planning**<br><br>In this theme, individuals seek assistance from ChatGPT-5 to plan age-appropriate leisure activities addressing physical, mental, and social needs specific to life stages (childhood, adolescence, adulthood, or old age).	
30	Can you suggest fun and developmental leisure activities for my 10-year-old child?	🎨 *Creative & Expressive Activities*<br><br>*Supports imagination, emotional expression, and fine motor skills*<br><br>• **DIY art projects** (paper crafts, clay modeling, recycled sculpture)<br>• **Story writing or comic book creation**<br>• **Make a "feelings journal"** with drawings and short entries<br>• **Build a puppet theater** and put on shows with friends or family

- **Create a vision board** using magazines and drawings

🏃 *Active & Physical Activities*

💪 *Builds coordination, endurance, self-regulation, and teamwork*

- **Obstacle courses at home or in the park** (pillows, cones, ropes)
- **Bike riding or scootering** with basic safety rules
- **Dance-offs or movement games** (Just Dance, freeze dance—no screens needed)
- **Beginner martial arts or yoga classes for kids**
- **Join a recreational team sport** (soccer, basketball, swimming)

🔬 *Exploratory & STEM-Inspired Play*

□ *Encourages problem-solving, curiosity, and science awareness*

- **Make a volcano or slime** using safe kitchen ingredients
- **Build with LEGO or magnetic tiles** using challenge cards
- **Grow a mini garden** (window herbs or vegetable seeds)
- **Do "kitchen science" experiments** (sink/float, density, baking reactions)
- **Simple coding toys or unplugged logic puzzles**

🎲 *Games & Structured Fun*

□ *Enhances focus, memory, patience, and social skills*

- **Board games** like Ticket to Ride, Qwirkle, or Uno
- **Memory and matching games**
- **Puzzles (jigsaw, word searches, or mazes)**
- **Create a scavenger hunt** (indoor or outdoor)

🌍 *Social & Outdoor Exploration*

□ *Boosts empathy, communication, and real-world learning*

- **Go geocaching or treasure hunting** with a paper map

- **Visit museums, nature centers, or zoos**
- **Have a weekly “friend day”**—host themed playdates
- **Volunteer together** (e.g., clean-up walks, animal shelter visits)
- **Start a “neighborhood explorer club”** with friends

📅 *Sample Weekly Leisure Plan (Balanced & Fun)*

Day	Activity	Developmental Focus
Monday	DIY art + story journaling	Creative + emotional
Tuesday	Outdoor obstacle course + picnic	Physical + planning
Wednesday	Kitchen science experiment	STEM + curiosity
Thursday	Board game night with family	Social + cognitive
Friday	Bike ride or team sport	Fitness + coordination
Saturday	Museum trip or nature hike	Discovery + attention
Sunday	Gardening + reflection drawing	Patience + calm focus

31

I’m a high school student and want to spend my non-study time more productively. How can I plan this?

🎯 *1. Define What “Productive” Means for You*

Productive free time isn’t just about academics. Think in **four categories**:

- □ **Skill-building** (learn something new)
- □ **Social connection** (friends, family, teamwork)
- 🎨 **Creativity and hobbies** (fun and self-expression)
- □ **Rest and renewal** (mental/physical well-being)

Ask yourself:

“What do I want to feel more of—confidence, calm, energy, inspiration?”
Your plan should reflect *you*, not just what others expect.

📅 *2. Weekly Leisure Plan (Flexible & Effective)*

Day	Activity Type	Example Activity
Monday	Skill-building	Watch a short tutorial (e.g., digital art, video editing, coding)

Tuesday	Creativity	Write a poem, journal, draw, or play music for 30 mins
Wednesday	Movement	Go for a walk, do a short workout or join a dance class
Thursday	Social/Connection	Call or meet a friend, volunteer, or join a school club
Friday	Fun & Play	Game night (offline or online), movie, or fun reading
Saturday	Personal Project	Work on a passion project (blog, science idea, small business)
Sunday	Mental Rest & Reflect	Digital detox + plan your week + light stretching or meditation

---

3. Try These High-Impact Leisure Activities

🚀 Skill-Building (1–3x a week)

- Learn graphic design (Canva, Procreate)
- Try beginner coding (Python, Scratch)
- Improve a language (Duolingo, or reading a novel)
- Join a competition or school club (debate, science fair)

🎨 Creative Expression (2–3x a week)

- Paint, doodle, or sketch
- Write fiction, blog, or spoken word poetry
- Try photography challenges with a phone
- Make music or remix tracks with free apps

Physical & Mental Wellness (Daily if possible)

- Yoga, walking, or a sport you enjoy
- Breathing exercises or journaling before bed
- Weekly nature time—without headphones

Social Growth

- Plan regular hangouts or study-with-friends sessions
- Practice conversation (great life skill!)
- Volunteer once a month—develops empathy and confidence

		✅ *Tips to Stay on Track Without Burnout*<br><br>• Use the **“2 out of 4” rule** daily: Choose 2 categories (e.g., social + skill)<br>• Plan with **mini time blocks**: 30 mins is better than none<br>• Keep 1 day as a *free day* to do anything you like—even nothing!<br>• Reflect weekly: *“What made me feel good? What do I want more of?”*
32	I’m a busy working adult. Do you have suggestions for quality leisure time in the evenings?	🌙 *Evening Leisure Ideas for Busy Adults*<br><br>□ ***If you have 15–30 minutes...***<br><br>□ **Mental Unwind**<br><br>• ✦ Light stretching + soft music<br>• ✦ Guided meditation or breathwork (try box breathing: inhale 4s, hold 4s, exhale 4s, hold 4s)<br>• ✦ Gratitude journaling: write 3 good things from today<br><br>📖 **Creative or Reflective Flow**<br><br>• ✦ Read a short story, article, or a few pages of a novel<br>• ✦ Doodle or sketch your day in images<br>• ✦ Write a letter to your future self (or just a brain dump)<br><br>---<br><br>⏳ ***If you have 30–60 minutes...***<br><br>□ **Learn Something You Love**<br><br>• ✦ Watch one documentary episode or TED-style talk<br>• ✦ Take an online mini-course (writing, photography, psychology, etc.)<br>• ✦ Practice a skill: guitar, a language, cooking a new dish<br><br>□ **Gentle Movement or Restorative Activity**<br><br>• ✦ Go for an evening walk (alone or with someone)<br>• ✦ Join a relaxing yoga or tai chi session (online or in person)

- ✦ Cook mindfully with slow music + candles

👥 Evening Social Options (once or twice a week)

- ✦ Invite a friend or partner for a tech-free dinner or tea
- ✦ Host a low-key “unwind night” (board games, music, shared reading)
- ✦ Attend a class you enjoy (dance, art, group meditation)

🗓 *Sample Weeknight Leisure Flow*

Day	Energy Level	Activity
Monday	Low	Journaling + tea + relaxing music
Tuesday	Medium	Walk + cook a new recipe
Wednesday	High	Creative hobby (music, painting)
Thursday	Low	Gentle stretching + mindful breathing
Friday	Medium	Social dinner or game night

🌟 *Tips to Make Leisure Stick:*

- 📅 **Schedule it** like a meeting—even 20 minutes matters
- 📵 **No screens** unless intentionally used (e.g., yoga video)
- 🌱 **Don’t aim to “achieve”—aim to feel** calm, joy, or inspiration

33	I’m retired, but my days feel empty. What activities do you recommend for an active life?	see below

🌟 *Holistic Leisure Activities for an Active Retirement*

☐ Mental Stimulation

Keeps your brain sharp, curious, and engaged.

- ✦ Join a **book club**, lifelong learning program, or local lecture series
- ✦ Do puzzles, crosswords, Sudoku, or memory games

- ✦ Learn something new: a language, an instrument, or digital photography
- ✦ Write your memoirs, a blog, or short stories

Physical Activity

Boosts energy, mood, mobility, and overall health.

- ✦ Daily walks (solo or with a walking group)
- ✦ Gentle fitness: yoga, tai chi, water aerobics, or stretching routines
- ✦ Gardening or outdoor work
- ✦ Dance classes or movement-based leisure (pickleball, bowling, golf)

🎨 Creative Expression

Sparks joy, flow, and self-discovery.

- ✦ Painting, sketching, knitting, or woodworking
- ✦ Join a local art or craft class
- ✦ Try photography, collage, or nature journaling
- ✦ Explore singing or learning a musical instrument

Social Connection

Maintains emotional health and community belonging.

- ✦ Host regular coffee mornings or potlucks
- ✦ Volunteer at a cause you care about (library, shelter, school, hospital)
- ✦ Join hobby-based clubs: birdwatching, chess, gardening, travel
- ✦ Take group classes or day trips with a senior center or local university

---

Mindful Leisure & Reflection

Balances stimulation with inner calm.

- ✦ Practice daily gratitude journaling
- ✦ Do light meditation or spiritual reading
- ✦ Spend time in nature: forest walks, stargazing, gardening
- ✦ Create a peaceful morning or evening ritual (tea, music, journaling)

🗓 *Sample Active Week (Flexible & Balanced)*

Day	Morning	Afternoon	Evening
Monday	Walk + crossword	Art class or craft time	Light reading or journaling
Tuesday	Garden or yoga	Volunteer at local center	Movie night (with friends)
Wednesday	Group walk or tai chi	Memoir writing or puzzles	Board game or card night
Thursday	Learn something new	Lunch with a friend	Relaxation + music
Friday	Photography walk	Cook a new recipe	Attend a local event
Saturday	Farmers' market trip	Home project or hobby	Call family or friends
Sunday	Nature time or rest	Reflect and plan week ahead	Meditate + gratitude list

---

✨ *Final Tips:*

- Don't aim to fill time—**aim to fulfill yourself**
- Alternate quiet and active days for balance
- Stay connected with community—**purpose often comes through others**

34	I can't find time for myself in family life. Can you suggest short	### □ *Short & Restorative Leisure Activities (10–30 mins)*<br><br>**□♀ 1. Breathing + Grounding Ritual (5–10 mins)**

	but effective leisure activities?	• Sit quietly. Inhale for 4 seconds, exhale for 6.<br>• Try it in a parked car, shower, or during nap time.<br><br>✅ *Benefit:* Calms nervous system, recenters your focus.<br><br>**📖 2. Read for Pleasure (10–15 mins)**<br><br>• Keep a novel, poetry book, or short essays in easy reach.<br>• Read 2–3 pages while sipping tea, in bed, or outside.<br><br>✅ *Benefit:* Mental escape + intellectual nourishment.<br><br>**🎧 3. Mini Sound Retreat (15–20 mins)**<br><br>• Listen to calming music, nature sounds, or an inspiring podcast.<br>• Close your eyes—or fold laundry while listening.<br><br>✅ *Benefit:* Reconnects you with your inner self or creativity.<br><br>**□ 4. Journal or Doodle Dump (10 mins)**<br><br>• Free-write or sketch what's on your mind—no rules.<br>• Try prompts like: *"What do I need right now?"*<br><br>✅ *Benefit:* Mental clarity + emotional release.<br><br>**🚶 5. Quick Walk or Solo Errand (15–30 mins)**<br><br>• Step outside without your phone (or keep it in your pocket).<br>• Focus on your breath, surroundings, or inner thoughts.<br><br>✅ *Benefit:* Physical relief + a dose of peace.<br><br>**☕ 6. "Do Nothing" Break (10 mins)**<br><br>• Sit with a hot drink and **do nothing**—no scrolling, no chores.<br>• Just breathe, stare out the window, or pet your dog.<br><br>✅ *Benefit:* Unstructured stillness refreshes a crowded brain.

<table>
<tr>
<td></td>
<td></td>
<td>

🎨 7. Tiny Creative Outlet (15–30 mins)

- Try watercolor, embroidery, collage, or a simple DIY craft.
- No goal—just play with color or texture.

✅ *Benefit:* Sparks joy and engages your senses.

📅 Bonus: Build a “Me Time” Habit

- Anchor one of these to an existing routine:
    - After putting kids to bed
    - While dinner is in the oven
    - Right after your morning coffee
    - In the bathroom with the door locked 😊

Even 10 minutes daily becomes powerful when it's **yours alone.**

</td>
</tr>
</table>
