# Supplementary material for: ChatGPT-5 as a leisure health advisor: multidimensional assessment of reliability, quality, usefulness and readability
Source: Front Public Health. 2026 Jul 30;14:1889768. doi: 10.3389/fpubh.2026.1889768 (PMC13469005; doi:10.3389/fpubh.2026.1889768)
Supplement: Supplementary file 2 [file Data_Sheet_2.PDF]

No	Questions	AB_ ChatGPT-5 _DISCERN	AB_ ChatGPT-5_GSQ	AB_ ChatGPT-5 Usefulness
1.	I get mentally exhausted during the day. Can you create a leisure plan to relax my mind?	2	4	6
2.	I have a stressful life. What leisure activities should I do to feel calmer?	3	3	6
3.	I struggle with focus. Can you recommend leisure activities to improve this?	3	4	6
4.	I feel burned out. I need leisure suggestions to support my psychological recovery.	4	4	6
5.	Can you create a leisure routine for me to mentally rest and renew myself?	3	3	5
6.	I constantly feel tense. Can you suggest relaxing activities I can integrate into my daily life?	4	4	6
7.	I want a more active lifestyle. What leisure activities could fit my daily routine?	4	5	6
8.	I'm too sedentary, and it bothers me. What leisure suggestions do you have to maintain my physical health?	3	4	6
9.	Can you plan fun leisure activities that incorporate exercise	4	4	6
10.	What leisure activities should I do regularly to stay healthy?	3	4	6
11.	I struggle with exercise. What alternative leisure activities can I try?	3	4	5
12.	I want to improve my stamina. Can you prepare a low-intensity but sustainable leisure plan?	3	4	6
13.	I feel emotionally drained. What leisure activities can balance my mood?	3	4	5
14.	I want to feel more at peace and explore myself. How should I plan my leisure time for this purpose?	4	4	6
15.	I'd like leisure suggestions that increase emotional awareness and nourish me.	4	4	6
16.	I want to rebuild myself. What supportive leisure activities could help in this process?	4	4	6
17.	Can you create a leisure plan with activities for my spiritual growth?	4	4	5
18.	I want to strengthen my social relationships. What types of leisure activities should I join?	3	4	5
19.	I want to meet more people. How should I spend my leisure time?	4	4	6

20.	Can you suggest socially-oriented leisure activities to reduce my loneliness?	4	4	5
21.	I want to spend time with family/friends. What activities could we do together?	4	4	6
22.	Can you plan leisure activities to develop my social skills?	4	4	7
23.	Could you recommend group activities or hobbies that encourage socialization?	3	4	6
24.	I'm constantly looking at screens. What leisure activities can I do for a more balanced life?	4	5	7
25.	I want quality time beyond digital games. What alternative leisure activities do you suggest?	3	4	5
26.	Can you create a leisure plan to balance my technology use?	3	4	5
27.	I'm looking for enjoyable activities outside the virtual world. What do you recommend?	3	4	5
28.	Can you plan physical/mental leisure activities to reduce my digital fatigue?	4	4	5
29.	I want a digital detox. Can you prepare a one-week plan for me?	4	4	6
30.	Can you suggest fun and developmental leisure activities for my 10-year-old child?	3	4	5
31.	I'm a high school student and want to spend my non-study time more productively. How can I plan this?	3	4	6
32.	I'm a busy working adult. Do you have suggestions for quality leisure time in the evenings?	3	4	5
33.	I'm retired, but my days feel empty. What activities do you recommend for an active life?	4	4	6
34.	I can't find time for myself in family life. Can you suggest short but effective leisure activities?	3	4	5
